# Kabuki Syndrome—Clinical Review with Molecular Aspects

**DOI:** 10.3390/genes12040468

**Published:** 2021-03-25

**Authors:** Snir Boniel, Krystyna Szymańska, Robert Śmigiel, Krzysztof Szczałuba

**Affiliations:** 1 Department of Medical Genetics, Medical University, Pawinskiego 3c, 02-106 Warsaw, Poland; snir.boniel@wum.onmicrosoft.com; 2 Mossakowski Medical Research Center, Department of Experimental and Clinical Neuropathology, Polish Academy of Sciences, 02-106 Warsaw, Poland; szymanska2@wp.pl; 3 Department of Paediatrics, Division of Propaedeutic of Paediatrics and Rare Disorders, Medical University, 51-618 Wroclaw, Poland; robert.smigiel@umed.wroc.pl

**Keywords:** Kabuki syndrome, KMT2D, KDM6A, mechanism, treatment

## Abstract

Kabuki syndrome (KS) is a rare developmental disorder principally comprised of developmental delay, hypotonia and a clearly defined dysmorphism: elongation of the structures surrounding the eyes, a shortened and depressed nose, thinning of the upper lip and thickening of the lower lip, large and prominent ears, hypertrichosis and scoliosis. Other characteristics include poor physical growth, cardiac, gastrointestinal and renal anomalies as well as variable behavioral issues, including autistic features. De novo or inherited pathogenic/likely pathogenic variants in the *KMT2D* gene are the most common cause of KS and account for up to 75% of patients. Variants in *KDM6A* cause up to 5% of cases (X-linked dominant inheritance), while the etiology of about 20% of cases remains unknown. Current KS diagnostic criteria include hypotonia during infancy, developmental delay and/or intellectual disability, typical dysmorphism and confirmed pathogenic/likely pathogenic variant in *KMT2D* or *KDM6A*. Care for KS patients includes the control of physical and psychomotor development during childhood, rehabilitation and multi-specialist care. This paper reviews the current clinical knowledge, provides molecular and scientific links and sheds light on the treatment of Kabuki syndrome individuals.

## 1. Introduction

Kabuki syndrome (KS) is characterized by distinct facial dysmorphism, growth retardation, psychomotor developmental delay and a wide spectrum of other manifestations affecting various body systems. Its genetic etiology has been proven approximately a decade ago [1]. Since then, KS has been increasingly observed in the family medicine and pediatrics setting. This paper reviews the current clinical knowledge, provides molecular and scientific links and sheds light on the treatment as well as quality of life of Kabuki syndrome individuals.

KS was first described in the early 1980s in Japan. It was named after the characteristic facial features which resemble the makeup of actors in the Kabuki theater in Tokyo. Initially, this syndrome was thought to be specific to the East Asian race; however, new data do not demonstrate clear prevalence of KS in any ethnic population. Total prevalence is estimated at 1 in 32 thousand [1,2]. The clinical diagnosis of KS often requires long-term monitoring because the phenotype changes over time—characteristic dysmorphism and other cardinal features tend to appear after several years of life. Prenatal, neonatal and family history is often unremarkable. Facial dysmorphism presence may be enough to raise suspicion of KS, after which genetic diagnostics must be performed to ultimately confirm KS. Usually, this is performed using Next-Generation Sequencing or Sanger Sequencing (more commonly in the past) [3]. These techniques aim to identify *KMT2D* or *KDM6A* pathogenic/likely pathogenic variants (the genes responsible for KS). Alternatively, Whole-Exome Sequencing can be used [4]. Even so, in some cases no genetic etiology is found in patients whose clinical picture is consistent with KS. In such patients, either an atypical clinical picture is concluded, or a different syndrome is diagnosed. Such cases are described in other parts of this review.

International consensus diagnostic criteria for KS were established in 2019. These aimed to facilitate the process of diagnosing KS. It was concluded that a definite diagnosis of KS can be made in patients of any age with a history of infantile hypotonia, developmental delay and at least one of the major criteria:

1. A pathogenic or a likely pathogenic variant in *KMT2D* or *KDM6A*.
2. Typical dysmorphic features including long palpebral features, eversion of the lower eyelid and two more of:
  a Arched and broad eyebrows, with notching or sparseness
  b Short columella and depressed nasal tip
  c Large, prominent or cupped ears
  d Persistent fingertip pads

A list of supportive clinical features was agreed upon, including short stature, microcephaly, cleft palate, lip pits, hearing loss, congenital heart defects, feeding difficulties and immunological disorders. A probable diagnosis can be made in a patient with a history of infantile hypotonia, developmental delay and at least three of the supportive clinical features, and a possible diagnosis can be made in a patient with two of the supportive clinical features. These criteria may need to be expanded in the future; however, the authors argue that their use should already be implemented in order to make the KS diagnostic process universal [5].

In the next chapters, we analyze the clinical characteristics of KS with regard to growth and endocrinology, neurology, cardiology, gastroenterology, laryngology, ophthalmology, oncology, nephrology and immunology. We also examine the effect of the KS molecular background on the clinical picture.

## 2. Molecular Genetics

In 2010, Ng et al. performed the sequencing of exomes of 10 unrelated patients with Kabuki syndrome (KS) diagnosed clinically based on a specific scoring system. They demonstrated that *KMT2D* variants underlie Kabuki syndrome [3]. *KMT2D* is also known as MLL2 (the latter name is considered outdated) [6]. Two years later, Lederer et al. described three patients with deletions encompassing the *KDM6A* gene on the X-chromosome. They claimed that *KDM6A*, which normally escapes inactivation, would be more likely expressed from an active X chromosome, so that the deletion of *KDM6A* would manifest itself in the affected individuals [7]. Miyake et al. then demonstrated that the point variants in *KDM6A* also cause KS. They examined 32 patients with KS diagnosed clinically who were negative for *KMT2D* variants. Two patients had nonsense *KDM6A* variants while one had a three base-pair deletion in *KDM6A* [8]. UTY is a homologue of *KDM6A* on the Y-chromosome. In the past, UTY was thought to have lost its demethylase activity and to be non-functional; however, knockout-mice models suggest that the gene is expressed in a cell. Female mice with homozygous *KDM6A* deletion do not survive, but hemizygous males do, as this is thought to be due to the UTY gene function. This suggests that UTY does play a role similar to *KDM6A* in metabolism and development [9].

The *KMT2D* protein is a histone (H3) lysine methyltransferase protein, while *KDM6A* acts as a histone demethylase. Both proteins have complementary functions. *KMT2D* is responsible for cell-type specific gene expression during differentiation. It functions to trimethylate histone-3-lysine-4, opening the chromatin and activating homeobox and NESTIN genes during cell differentiation. *KDM6A* demethylates histone-3-lysine-27, closing the chromatin. Both genes affect the opening of chromatin and promote gene expression. Dosage sensitivity may be important in Kabuki syndrome. The switch between open and closed chromatin affects the access of transcriptional machinery to target genes, which may lead to the development of the disease [10]. Pathogenic/likely pathogenic variants seen in KS tend to reduce *KMT2D*’s catalytic activity. This in turn leads to an increase in methylated histone levels, which decreases homeobox gene expression, thus affecting global cell differentiation [11]. Building on this microbiological knowledge, Lee et al. demonstrated that a deletion in *KMT2D* decreases mediator and polymerase levels, which leads to defects in cell-type specific gene expression and cell differentiation. *KMT2D* was identified as a mono and di-methyltransferase, which is essential for enhancer activation during cell differentiation [6]. Drug-induced balance-restoration that promotes open-chromatin (for example, with histone-deacetylase inhibitors) may be considered as a novel KS treatment. Drug administration in mice normalized structural and functional deficits in dentate gyrus function after H3K4 trimethylation [10].

In humans, the *KMT2D* gene is located on chromosome 12. It is over 19 kilobase pairs in length and contains 54 exons. *KMT2D* codes for the enhancer histone-3-lysine-4 methyltransferase protein, one of six KMT proteins working as part of a chromatin modifier protein complex. Enhancers aid in gene expression regulation. They are often bound to transcription factors. *KMT2D* selectively binds to specific regions during various cell differentiation stages, activating gene expression depending on the differentiation stage [12]. A recently published Xenopus frog model provides evidence that *KMT2D* loss-of-function variants inhibit neural crest development, suggesting that KS be considered a neurocristopathy. The expression of *KMT2D* in the neural crest during pre-migratory as well as migratory stages was found. *KMT2D* may be required for neural crest cell differentiation and consequent migration [13]. Cell-autonomous proliferation and cell cycle defects along with early neural stem cell progenitor maturation in an in vitro and mouse model were confirmed. *KMT2D*-caused transcription suppression led to the activity of hypoxia-response pathways. This proves that the loss of *KMT2D* function suppresses oxygen-responsive gene programs crucial for neural progenitor maintenance, ultimately leading to precocious neuronal differentiation [14]. A recent zebrafish model aimed to analyze the role of *KMT2D* and *KDM6A* in the development of tissue abnormalities, including craniofacial, heart and brain deformities. Variants in both *KMT2D* and *KDM6A* lead to improper cell differentiation, ultimately causing a characteristic dysmorphism and developmental delay [15]. A fluorescence in situ hybridization and Whole Genome Sequencing study was performed, analyzing a female KS patient with a known *KDM6A* variant and a phenotype including hypotonia, developmental delay, short stature, microcephaly, seizures, facial dysmorphism and cleft palate. *KDM6A* expression is significantly reduced in neural crest cells, leading to delayed neural development. The dosage sensitivity of *KDM6A* is associated with characteristic Kabuki syndrome dysmorphism.

*KMT2D* and *KDM6A* proteins regulate the differentiation of mesenchymal cell lineages: myoblasts and osteoblasts. Mouse models have shown that lysine demethylase plays a crucial role in regulating mesenchymal lineage cell differentiation. Loss-of-function variants in lysine demethylase genes cause improper development of cells in this lineage and, presumably, several clinical neurological abnormalities that have been seen in KS patients. The anomalies present in KS children were also observed in mice with a loss-of-function *KMT2D* variant and brain development disorders: hypotonia, enhanced activity (corresponding to hyperactivity in KS children), a decreased auricular reflex (corresponding to hearing impairment) and motor coordination dysfunction [16]. *KMT* and lysine demethylase proteins’ specific role in the regulation of myoblast differentiation was also recently examined using the *C2C12* myoblast model system. *KDM1A* is the only one crucial for myogenic differentiation, while *KDM6A* (among others) is required for osteoblast differentiation. *KDM1A*, via histone-demethylation, represses the regulator of osteoblast differentiation, thus promoting myogenesis. *KMT2D*, however, is also required for osteoblast differentiation by the demethylation of a different receptor. These mechanisms of histone modification (lysine methylation) act as a signal, thus impacting the fate, rate and process of cell differentiation [17].

The effect of the deletion in the *KMT2D* gene of the region encoding for methyltransferase (thus leading to the impairment of its function) resulted in impaired histone acetylation and methylation, eventually leading to a deficiency of the dentate gyrus granule cell layer and to reduced neurogenesis and hippocampal memory defects. A deficiency in postnatal neurogenesis directly results in the *KMT2D* defect as the base for intellectual disability in KS [10].

*KMT2D* variant demonstrated sensory and psychomotor impairments, including hypotonia and impaired reflexes (suggesting impaired neurological function) and a reduction in the auricular reflex (suggesting hearing impairment) in mouse mutants. Increased general activity with decreased motor coordination, increased rearing and grooming and increased distance traveled and speed were observed. This study builds on Bjornsson et al.’s findings by presenting the *KMT2D* variant phenotype as more complex. This mouse model may shed light onto the wide range of psychomotor and behavioral impairments associated with KS in humans [16].

Missense *KMT2D* variant s likely cause reduced histone methylation due to impaired WRAD protein complex formation [18]. They may result in a novel multiple-malformation syndrome [19]. Notable clinical differences may be observed: no patient with such variant s presented with intellectual disability, clefting, renal abnormalities or seizures, while six patients presented with hypothyroidism. Missense variants may cause a disturbance in the *KMT2D* secondary structure through increased alpha-helical structure (coiled protein shape leading to dysfunction).

## 3. Growth and Endocrinology

Growth pattern in the pediatric population is dependent on several factors. These can be divided into intrinsic factors and external factors. Intrinsic factors (influenced by genetic factors) largely result from constitutive and familial growth or the presence of major congenital anomalies. External factors are shaped by prenatal care, labor mode, adaptation period, hormonal characteristics, feeding and nutrition during the first year of life and later into adolescence. On top of that, syndromic diagnosis by itself seems to carry important contributions to the growth pattern. Early observations of physical characteristics in Kabuki syndrome by Niikawa et al. suggested that birth weight and length were normal with ensuing postnatal growth retardation [20].

KS patients commonly present with failure to thrive during infancy, feeding problems and reflux, with 65–74% of patients even requiring nasogastric tube insertion or gastrostomy. Conversely, during late childhood these children commonly present with obesity. Awareness and intervention by a dietician should be considered [2].

Postnatal growth retardation was present in 50–70% of patients described in ensuing studies. Prenatal growth retardation was observed in a minority of analyzed cases and postnatal growth retardation was observed in the absolute majority, regardless of ethnic origins. [21]. In a review of 300 patients, birth weight and length were generally normal, with significant growth delay starting as early as the first year of life. Short stature was present in 75% of patients. This is consistent with previous reports of mean heights of over two standard deviations below average. Growth hormone deficiencies are present in a few cases [22].

KS children are generally born at term and with a normal birth weight. In one study, not one individual had a birth weight under the 3rd centile. Failure to thrive and feeding problems are observed in two thirds of patients. During the fifth year of life, progression to obesity is prevalent in up to 57% of patients [23].

Up to 70% of KS patients present with short stature. This has been suggested to be directly caused by the *KMT2D* variant [4]. Genotype-phenotype correlations revealed a significantly higher frequency of short stature in patients harboring a *KMT2D* variant than in those without it (*p*-value < 0.0001). Patients with a mosaic *KMT2D* variant less frequently presented with short stature. Growth Hormone -deficiency is the most common endocrinological finding in KS patients. Endocrinological evaluation and even the possibility of rh-GH-therapy for KS-patients may be beneficial [4].

Indications for rh-GH-therapy in pediatric patients include confirmed GH-deficiency, Chronic Kidney Disease, Turner syndrome, Intrauterine growth restriction/Small for gestational age infants who fail to catch up to normal growth centiles, Prader-Willi syndrome, idiopathic short stature, SHOX gene haploinsufficiency and Noonan syndrome (USA) [24]. Genetic syndromes without GH-deficiency (as in many cases of KS) can be treated with rh-GH-therapy as a trial.

The largest study to date by Schott et al. in 2017 analyzed the effect of growth hormone treatment on linear height and body proportions in KS patients. The result was a statistically significant linear height increase after one year’s worth of rh-GH-therapy. Children who received GH-treatment at a younger age tended to display greater height increases than those who started at an older age. No significant difference was described between the *KMT2D* versus the *KDM6A* group. Additionally, throughout the treatment course, patients’ body proportions were not affected [25].

Normative growth curves especially designed for children with KS based on the largest cohorts to date were recently created. They reaffirmed observations by Schott et al. in 2017 that *KMT2D* pathogenic variants showed growth patterns roughly two standard deviations below the norm, enabling growth monitoring using growth charts. It is crucial not to miss a separate non-syndromic etiology of disrupted growth (e.g., GH-deficiency, which can be observed in 2–22% of KS individuals). The limitation is that it is difficult to predict whether final KS patient growth rate will catch up to that of children not affected by KS. The normal distribution of Body mass index BMI in KS patient groups is much wider than in the general population, which suggests the increased presence of excess weight or even obesity in KS patients [26]. This obesity has been observed starting at the age of 4. This is consistent with findings in other studies [23,27].

There is support in the literature for evidence of a link between molecular genetics mechanisms and growth pattern in KS. First, a balanced interplay between gonadal and adrenal steroid hormones and GH is essential for the pubertal growth spurt. Patients with a deficiency in any of these hormones experience a deficient growth spurt.

During puberty, estradiol promotes the epiphyseal fusion essential for bone growth and thus directly affects adult height in both sexes. Ligand-dependent estrogen-receptor-α acts as a mediator in estrogen-sensitive tissues. Molecularly, *KMT2D* is essential for a ligand-dependent estrogen-receptor-α activation [28]. More details are in the *Molecular Genetics* section.

Decreased growth associated with decreased serum levels of Insulin-like growth factor-1 has been described in mouse models. The GH to IGF-I axis is highly active during puberty. The effect of estrogen on this axis has been theorized to happen through the direct action of testosterone/androgen receptors, or through the indirect action of estrogens on estrogen-receptor-α. This suggests a biological mechanism for a significantly diminished pubertal growth spurt in KS. Finally, testosterone stimulates GH-secretion in puberty and throughout adulthood. This effect depends on the level of aromatization of testosterone within estrogen [29]. Perhaps a *KMT2D* variant causes a decrease in the GH/IGF-I axis activity, and therefore a diminished growth spurt and short stature.

KS mice phenotypes are comprised of skeletal growth retardation and decreased length and weight as a cause of shortened long bones [30]. These findings are also seen in KS patients [31]. The *KMT2D* and *KDM6A* gene variant influences endochondral ossification at long bone growth plates by disrupting the estrogen receptors in developing skeletal tissues. Sex-based differences are not only dependent on hindered estrogen receptor activity as a result of gene variant. SOX9 has been linked to the pathogenesis of the KS phenotype by describing disrupted endochondral bone formation. In theory, *KMT2D* allows SOX9 to become uninhibited, thus activating chondrocyte differentiation (SOX9 being a well-described chondrocyte-differentiation mediator). Variants in *KMT2D* prevent this process from taking place. SOX9 has also been described as involved in inhibiting osteoblast differentiation. Abnormal SOX9 expression (for instance, as a result of *KMT2D* variant) prevents the differentiation of chondrocytes into osteoblasts, ultimately causing too many chondrocytes and too few osteoblasts in the skeletal system. This probably causes improper growth, specifically growth retardation in KS (shortening of long bones and ineffective bone formation) [30].

A wide variety of hormonal abnormalities can occur as a result of KS. Kabuki syndrome has been recently associated with hyperinsulinism and thus to transient neonatal or infantile hypoglycemia. Persistent hypoglycemia, however, is uncommon. KS patients with a *KDM6A* variant have an increased risk of hyperinsulinism (and thus hypoglycemic episodes) than those with *KMT2D* pathogenic variants [32]. *KDM6A* demethylation of the H3K27 protein may lead to the deregulation of β-cell development [33]. *KDM6A* codes for a demethylase that acts on H3K27me3/me2 and induces a steady-state in proliferating cells [34]. Most actively these domains increase in number within endocrine pancreatic cells, which leads to disorders of pancreatic β-cell development.

It is hypothesized that KS is associated with excess gonadotropin secretion, which causes premature thelarche in 41% of patients [2]. One patient exhibited delayed thelarche (constitutional delay of puberty) along with other endocrine symptoms (obesity, congenital hypothyroidism and GH-deficiency). This may be rooted in estrogen-receptor downregulation as a result of KS. The majority of patients present with short stature as a direct result of GH deficiency [2]. Additionally, KS has also been linked to combined pituitary hormone deficiency, rooted in the deletion of polyalanine tracts. One Japanese male KS patient with combined pituitary hormone deficiency was reported. Genetic tests identified a novel hemizygous 21-base pair deletion in this patient, resulting in the loss of seven alanine residues from the polyalanine tracts in the SOX3 gene. This suggests that deletions in the polyalanine tracts can cause hypopituitarism in these patients [35].

## 4. Neurological Issues in Kabuki Syndrome

### 4.1. Foreword

Loss-of-function variant s associated with Kabuki syndrome (KS) lead to the dysregulation of the differentiation of neuronal and myogenic cells. Mechanistically, this may lead to several neurological abnormalities in KS patients. These include infantile hypotonia, developmental delay and intellectual disability, epilepsy, behavioral abnormalities including autistic-like features, and CNS malformations. Neuropsychological symptoms have more commonly been observed in patients with *KMT2D* variant s [2]. It is important to note that unintelligible or disordered speech originating in oral motor hypotonia has consistently been reported throughout the literature and is commonly observed by the parents and other caretakers [36].

### 4.2. Infantile Hypotonia

Infantile hypotonia has been described as a cardinal feature in KS patients [37]. Specifically, oral hypotonia can present as difficulty in the ability to suck, chew and swallow. Delayed psychomotor development in the first two years of life may also be present. KS children between the ages of 3 and 6 years characteristically present with open mouth at rest [38]. Up to 90% of KS patients between the ages of 6 and 14 years present with ligamentous laxity, mainly of the shoulder, hip and knee joints. Sometimes, this symptom can disappear with age and following early rehabilitation [39]. During intubation before large procedures under general anesthesia, excessive ligamentous laxity may lead to instability of the cervical spine. Proper positioning before anesthesia is crucial [39]. Growth hormone therapy may help reduce the severity of excessive joint laxity in KS. In a recently examined cohort of 27 KS patients with diagnosed large-joint hypermobility, there was a statistically significant decrease in the presence of joint hypermobility after 2 years of growth hormone therapy [40]. Gross motor developmental milestones are delayed in KS. In the absence of structural brain anomalies, developmental delay is at mild to moderate degree in KS. The age of unassisted sitting ranges from 7 to 19 months, walking age from 15 to 30 months, while the single word acquisition age is from 10 to 30 months. The presence of associated abnormalities, for example, hearing loss, does not seem to directly affect motor development. These children also perform weakly on cognitive tests, namely due to attention deficit associated with the developmental delay [41,42].

### 4.3. Developmental Delay

It has been suggested that developmental delay is more pronounced in patients with *KMT2D* gene deletions or variants causing a dysfunction of synthesis in the first half of the *KMT2D* protein [43]. However, no specific genotype-phenotype correlations could be drawn [43]. Varying degrees of developmental delay and speech disability are present in all individuals with KS. Psychomotor retardation or intellectual disability (mean intelligence quotient of 35–69) occurs in all children with KS. Children with KS tend to have a particularly deficient visual memory and visual-spatial coordination [42]. However, they are distinguished by a relatively broad functioning vocabulary capability. Disordered speech is, in fact, an important issue in children with Kabuki syndrome, typically presenting with a dull, flat tone or an increased inflection pattern in speech, stemming from the characteristic oral hypotonia associated with Kabuki syndrome. Articulation errors led to impaired speech intelligibility. Pitch, loudness and prosody did not mature with time, suggesting that speech was significantly delayed in these patients [39,42]. There are 31 KS patients in whom the correlation between visual deficiency as well as the presence of a truncating variant and a resulting reduced IQ has been described [44].

### 4.4. Neurobehavioral Abnormalities

Several studies [43,44,45,46] describe neurobehavioral findings in the context of KS. These may include learning disabilities with reduced IQ, adaptive skill impairment, autistic-like behavior and psychiatric pathologies, such as anxiety disorder, phobias, bizarre behavior and impaired emotional control.

Autistic-like behaviors described in KS cases included: preference to be alone, selective interest in specific objects, stereotype behavior, attention instability and echolalia. Upon analysis using the CARS, ADOS and ADI-R scales (used in autism spectrum disorders), patients tended to fulfill the criteria of autism. They generally had low IQ. Some did present abnormal neurological findings; however, these did not seem to directly affect autism spectrum behaviors. It has been suggested that genetic variants that cause KS promote the development of autistic-like behaviors through epigenetic mechanisms, as in other diseases such as Rett syndrome, Fragile X syndrome, Prader-Willi and Angelman syndrome [46]. Thus, therapy for autistic-like behaviors should focus on the capacity of understanding (receptive communication). The clarification of physical environment (association of each place with a single activity) may help develop these children’s behavioral understanding. Regularity and consistent scheduling of times and activities may aid in these patients’ development, as a therapy for the autism-like behavior and an improvement of quality of life [46,47].

Three patients with a mosaic *KMT2D* variant —a rare phenomenon in KS patients—have been described. Systemic features in mosaic KS patients are not significantly different from those of non-mosaic patients. The difference was seen in psychiatric manifestations: anxiety disorder, multiple phobias, bizarre behavior (soliloquy), emotional dysregulation and autistic-like behavior [43]. The intelligence and cognition profiles of KS children were analyzed. Even though children in the cohort presented a wide range of intellectual abilities, most children had only a moderately impaired IQ. Individuals in their study tended to have better abilities in working memory and vocabulary comprehension than speed processing or perceptive reasoning. It was also found that some features can potentially modify IQ in KS patients. First, girls tended to score better than boys on IQ testing. At present, no specific cause for this is put forward. While analyzing Perceptual Reasoning Index scores, it was noticed that KS children fared comparably to their healthy peers until the age of 10 years. The decrease in skill acquisition rate at this age can be traced down to schooling technique. Conversely, organic malformations did not affect IQ, unlike in previously conducted studies [44].

A novel approach refers to IQ as a memory deficit in the context of KS. Histone deacetylase inhibitor protein HDACi-AR-42 affects hippocampal memory by improving adult neurogenesis [48]. Building on this finding, the ketogenic diet has been shown to improve cognitive function in KS patients. Ketogenic diets lead to the release of beta-hydroxybutyrate (an energy-rich product of lipid metabolism released upon hypoglycemia during ketogenic diet). Beta-hydroxybutyrate is an endogenous histone deacetylase inhibitor released from hepatic cells. In theory, more effective histone acetylation thanks to beta-hydroxybutyrate activity on a ketogenic diet leads to a more effective neuronal development (the study focused on the hippocampus). A mouse model demonstrated an improvement in hippocampal memory function and the mechanism behind it. Ketogenic diets act as a dietary intervention that, unlike a drug, can be quickly implemented clinically. It is an epigenetic mechanism which affects transcription in neurons as well as nuclear structure, which can be seen clinically [48].

### 4.5. Epilepsy

The prevalence of epilepsy in patients with Kabuki syndrome has not been accurately established. Some studies suggest it is close to 5–16%, while other estimations are up to 36%. Seizures may result from infantile hypoglycemia or arise from abnormal electrical activity within the brain [49]. A range of seizure types has been described. These most commonly include focal seizures, convulsive seizures and myoclonus. Epilepsy resulting from abnormal electrical activity within the brain is partially responsive to conventional therapy. Epilepsy is present in 36% of tested patients (five individuals, of whom four had nonsense variants and one had a missense variant)—higher than previously published reports. Myoclonic seizures tend to respond well to levetiracetam, while focal seizures tend to respond poorly to carbamazepine therapy. Evidence of brain malformation since birth was not shown; however, 4/5 of these children had abnormal electroencephalogram findings. The age of seizure onset was relatively advanced (median 11.8 years). This corresponds to the case reported by Bekircan-Kurt et al. They conclude that, bearing in mind the relatively late age of onset, seizure frequency increases with age. Antiepileptic drug therapy effectively controlled seizures and EEG tracings in most patients with epilepsy on follow-up [50,51,52].

Four case reports describe patients with brain anomalies on imaging (i.e., polymicrogyria and generalized epilepsy with favorable response to medication; gray-matter nodular heterotopia with dysgenetic corpus callosum; perisylvian polymicrogyria; and perisylvian cortical dysplasia). This suggests that brain development and, more broadly, findings upon brain imaging are variable in the context of KS [53,54,55,56]. Seven KS patients presented with homogenous EEG findings characterized by isolated or repetitive biphasic spikes or sharp waves, followed by a slow wave of medium and high voltage in the fronto-central regions, probably leading to focal seizures [57]. A boy with a novel nonsense *KMT2D* variant presented with characteristic KS dysmorphism as well as complex partial seizures. The seizures were partially responsive to antiepileptic drugs. Exon 39 of the *KMT2D* gene contains a region that encodes for a long polyglutamine chain. This chain is sensitive to variants which may cause CNS and other organ dysfunction [58]. KS has been rarely associated with Chiari type I malformation (caudal herniation of the cerebellar tonsils through the foramen magnum). Symptoms associated with this malformation are nonspecific and include chronic headaches, especially of the suboccipital region, exacerbated by coughing or straining, neck pain, upper limb weakness and pain, vertigo, dysphagia with vomiting and late-presenting gait abnormalities (ataxia). The possibility of the Chiari type I malformation presenting more frequently in the context of specific neurologic and genetic syndromes is currently under investigation. In rare cases, Chiari type I malformation was diagnosed in adolescent KS patients. It has thus been suggested that it may be present in KS patients more frequently than previously reported. Importantly, it highlights the importance of brain imaging and the early recognition of seizures in this group [59,60].

Other rare single case reports of neurological abnormalities in the context of KS have been described. KS patients often present with nonspecific cerebral atrophy and microcephaly. CNS imaging is heterogenous in KS. A child with a confirmed *KMT2D* pathogenic variant presented with microcephaly and craniosynostosis [61], which may be included as a component manifestation of the KS phenotype [62]. A KS patient with recurrent immune thrombocytopenia and recurrent infections due to immunodeficiency was treated for status epilepticus in the intensive care unit. Brain magnetic resonance imaging MRI showed a frontal lesion. Upon biopsy of this lesion, diffuse polyclonal lymphoplasm cellular cerebral infiltrate with perivascular inflammation was found. This lymphocytic infiltrate of the brain could be directly caused by *KMT2D* variants and should be suspected in the context of KS even when facial dysmorphism is not present [63]. An adult woman with KS presented with tremor and epilepsy and brain nuclei hyperintensities upon brain imaging. A KS patient with sudden-onset chorea with positive serum antiphospholipid antibodies supports the hypothesis of the increased risk of immune dysregulation in the context of KS (see Immunology chapter) [64]. Together, this group of rare KS case reports presents the wide variety of CNS manifestations in this syndrome. Besides epilepsy, CNS symptoms commonly present in KS patients are sensorineural hearing loss (also see Otolaryngology chapter), chorea, developmental/psychomotor delay and neurobehavioral abnormalities. Organic brain abnormalities, such as a decreased volume of grey matter, decreased cerebral blood flow, among others, to the hippocampus, are also present. This suggests a neural basis for the cognitive impairment observed in KS [65,66].

Most importantly, early intervention and rehabilitation have been described as beneficial for both patients and their caretakers. This is especially true if a collaborative treatment team of doctors, teachers, physical therapists, speech therapists and psychotherapists actively work in concert with the family. Specific psychotherapy techniques for KS patients should be considered on a case-by-case basis [67].

## 5. Cardiovascular Issues in Kabuki Syndrome

Cardiological manifestations are cardinal features of Kabuki syndrome. The three most commonly observed cardiac defects in KS patients are atrial septal defect, (ASD), ventricular septal defect (VSD) and coarctation of aorta (CoA) [2]. An analysis of specific congenital heart disease (CHD) manifestations in a 60 patient cohort was conducted. CHD was diagnosed in 58% of them, mostly in males. While analyzing for specific CHD, significant male predominance was observed in CoA cases, while no sex-related predominance was observed in VSD patients [68]. Rarer cardiac defects include aortic stenosis, pulmonary stenosis, tetralogy of Fallot (ToF), double-outlet right ventricle, complete transposition and Ebstein’s anomaly [69].

Up to 80% of KS patients with *KMT2D* variants present with CHD [70]. CoA is often described in the literature in the context of KS. Juxtaductal CoA is the most common subtype observed in KS patients, sometimes considered a cardinal feature of KS [71]. CoA may be present with or without [69] other heart defects such as anomalous left pulmonary artery [72] or left ventricular diverticulum [73]. Taking the prevalence of different CHD types into account, a predominance of left-sided obstructive lesions can be observed (nearly half of all patients), e.g., Shone complex type of anomalies [70]. Together, aortic coarctation and septal defects are the most common CHD in patients with *KMT2D* variants (nearly half of patients). Of septal defects, VSD and ASD-II are predominant. Bicuspid aortic valve has been reported in 1/8 of patients. Mitral stenosis, hypoplastic left heart syndrome, conotruncal defects, cardiomegaly, tetralogy of Fallot and other rare syndromes have been reported in under 5% of patients [70].

*KMT2D’s* role in cardiogenesis has been demonstrated in mice. *KMT2D* deletions lead to decreased histone-3-lysine-4 methyltransferase expression at enhancers and promoters of cardiogenesis. This results in the downregulation of ion transport and cell cycle genes. Moreover, *KMT2D*-bound regions within cardiomyocytes have been defined [74]. Variants cause decreased gene expression in *KMT2D* knockdown mice hearts [74]. Disorders of ion transport, hypoxia-reoxygenation and cell cycle regulation were observed. While analyzing the development of congenital cardiac abnormalities, a single functional copy of *KMT2D* may be enough for normal heart function. A deletion of both copies leads to general disruption of myocardial development [74]. On the contrary, a newborn with a de novo *KMT2D* heterozygous frameshift deletion presented with VSD as well as a myriad of other cardinal KS features. Good general condition and prognosis was seen, and surgical correction of cardiological abnormalities was performed. This highlights the importance of a case-by-case surgical treatment approach to KS patients as well as genotype-phenotype-driven diagnostics [75]. Knockdown *Xenopus* frogs develop hypoplastic hearts with defective chamber development. Cardiomyocyte differentiation is severely affected by a loss of *KMT2D* function. *KMT2D* is required for the development of primary and secondary heart fields [76]. A link between the Notch pathway and *KMT2D* during endothelium and endocardium development has been suggested in zebrafish. Notch pathway inhibition leads to physiological cardiovascular development. Extrapolating on this finding, *KMT2D* may play a role in regulating vasculogenesis and angiogenesis (connective tissue development) [77]. Yet, *KMT2D*’s function as a cardioregulatory gene in humans is not yet fully understood, nor is its role in the development of specific congenital cardiac diseases [74,76,77]. Fewer than half of patients with *KDM6A* variants present with CHD, and those who do show mostly right-sided lesions [70]. Mouse models presented with abnormal atrial and/or ventricular development and myocardial wall bulging defects in heterozygous as well as homozygous subjects [15,70].

Surgery is commonly indicated to treat congenital cardiac anomalies in KS patients. Usually, KS patients tolerate the surgical correction of CoA well, and cardiological prognosis is positive [71,73,78]. Similarly, ASD may be repaired using minimally invasive robotic techniques, further improving KS patient postoperative quality of life [79]. Indications for surgical intervention depend on the general condition and prognosis of the child. No special considerations based on *KMT2D* variants have been suggested [75].

Underlying connective tissue diseases may ultimately lead to cardiac defects in KS patients. Connective tissue developmental disorder in the context of KS has been proposed for decades, mostly while describing hip, knee and shoulder joint hypermobility in patients with *KMT2D* variants [2,80]. Digilio et al. discuss clinical overlaps between KS male patients and Turner syndrome patients since CoA is the most common congenital heart defect present in Turner syndrome [68]. Perhaps a similar pathomechanism leads to the development of CoA in KS patients. KS patients may be predisposed to express connective tissue abnormalities. The management of these anomalies is conservative because, when compared with anticoagulation therapy, no difference in effectivity has been demonstrated [80]. Great vessel pathologies, including aortic and/or main pulmonary artery aneurysms [81] and double aortic arch [82], may arise from connective tissue disease associated with KS. These are usually successfully managed with aortoplasty [81].

Systemic vascular abnormalities in the context of KS have been recently reported. Gatto et al. describe a case of a KS patient who suffered a transient ischemic stroke due to occlusion of the right internal carotid artery. Despite the fact that cardiac and great vessel anomalies are common (as we demonstrate above), some rarer systemic vascular anomalies have been reported too, affecting among others small vessels of the brain [83]. It is important to consider vascular anomalies as risk factors for ischemic disease as well as stroke [83].

Besides conotruncal, great vessel or connective tissue anomalies, an association of left-sided heart anomalies with KS has been theorized. The presence of normal X chromosomes can prevent the development of left-sided anomalies. Left-heart anomalies are of multifactorial origin [84]. KS patients with hypoplastic left heart syndrome (HLHS) tend to present with more severe cardiac symptoms than those with shunt anomalies. These may include failure to thrive and feeding difficulties. Prognosis is poorer than that of patients with shunt lesions. Patients typically undergo palliative surgery aimed only at improving quality of life, since the underlying disease cannot be corrected (unless cardiac transplantation is considered) [78,85]. Left-heart anomalies may shorten the lifespan of KS individuals.

One rare case of a child with KS, who died of untreated arrhythmia at the age of 11 months, was reported. The arrhythmia resulted from a cardiac conduction abnormality causing episodes of bradycardia unresponsive to pacing, which eventually led to asystole and death. On top of that, the child suffered from severe immunodeficiency. Autopsy revealed coronary sinus dilatation, tricuspid valve dysplasia, thickened chordae tendinae and right ventricular hypertrophy. Conduction abnormalities in KS children have not been extensively investigated [86].

Most reports do not describe severe symptomatic heart disease in KS patients. In fact, some KS patients have remained cardiologically asymptomatic, or their symptoms have been masked by the underlying disease (such as in the case of congenital heart defects described by Digilio et al. [68]). Thus, when considering the quality of life of KS patients, it is important to emphasize early recognition and treatment of cardiological abnormalities. Echocardiography should be performed in all the patients at the time of diagnosis in order to enable early recognition of structural cardiac anomalies [70]. In patients with *KMT2D* variants, attention should be paid to the detection of left-sided obstructive lesions, while in patients with *KDM6A* variants, right-sided lesions should be the subject of attention [70]. Individuals with aortic anomalies should be monitored at least annually for aortic dilatation [82]. All patients with structural cardiac anomalies should be referred to a cardiologist [70]. If treatable, appropriate therapy and recovery from cardiac disease will enable effective treatment directed at other ailments associated with KS in order to improve quality of life.

## 6. Gastrointestinal (GI) Issues in Kabuki Syndrome

Kabuki syndrome patients frequently present with poor feeding and nutrition, which leads to poor growth. They often have sensory issues that interfere with eating, rendering them more sensitive than other children. Textures, smells and temperatures of food may cause these children to present with aversions. Due to poorly coordinated suck and swallow reflexes, a nasogastric (NG) feeding tube or even a gastrostomy may be required [87,88]. Reflux tends to be more common and more severe in the KS population [87]. Severe reflux can present with recurrent aspiration, eventually culminating in oxygen-dependency, chronic lung disease and death due to complications of neuromuscular and pulmonary involvement [87]. At later ages, feeding therapy should be recommended for parents in order to educate these children to an encouraging mealtime experience and to improve these children’s eating habits. Proper feeding and nutrition is crucial for quality of life improvement [87,88].

Gastrointestinal (GI) anatomical dysmorphism has been described in single KS cases too, especially in females. It has even been suggested that KS in females is a risk factor for anorectal malformations [89]. KS individuals may present with intestinal malrotation, anal atresia, anovestibular fistula or anterior anus. Lower GI anomalies are more likely in KS patients and generally have a positive prognosis, given that the management of these abnormalities in KS patients is not different from that of nonsyndromic patients [89]. KS should be initially suspected clinically in female patients with characteristic dysmorphism and anorectal malformations. Pediatric surgeons should be aware of the potential for serious cardiac defects in these patients [89]. These suggestions are, however, only based on two cases. Nevertheless, they can positively impact these patients’ quality of life thanks to early recognition. KS children may be at increased risk for pancreatic ductogenesis abnormalities, leading to pancreatitis. The specific cause of abnormal pancreatic duct morphology and pancreatitis is unclear, but may result from cytochrome C deficiency and/or abnormal enzyme production [90]. There may be an increased risk of esophageal or gastric polyp development—a phenomenon occurring in 0.1% of pediatric patients. These polyps are usually symptomatic at onset, causing heartburn, nausea and vomiting. Treatment should be individualized and may include proton pump inhibitors and, in case of lack of improvement, endoscopic polypectomy to relieve symptoms [91].

Hepatobiliary abnormalities, biliary atresia, hepatic fibrosis and sclerosing cholangitis have been documented in 2–21% of KS patients [92]. Very little information is available regarding long-term follow-up of these patients’ liver disease. Liver transplantation in young adulthood may be the only way to overcome underlying liver disease, though severe progression may be preventable thanks to early recognition. Sclerosing cholangitis and dyslipidemia may be successfully treated with ursodiol and cholecystyramine [92]. The specific etiology, pathophysiology and hyperbilirubinemia clinical course in these patients in the context of KS remains unclear, because even though liver disease is described in KS, there appears to be no common factor between cases [92]. Liver disease may have an immunological basis, especially considering that KS patients often suffer from other autoimmune diseases. In such cases, based on findings by Suskind et al., ursodiol in combination with cholecystyramine should be considered in KS patients who suffer from cholestatic disease. When possible, using these drugs significantly improves quality of life and may, in theory, delay or even cancel liver transplantation. Primary liver tumors (namely hepatocellular carcinoma), though rare in children, may be at risk for development in KS patients due to the underlying condition. *KMT2D* has been found to be somatically mutated in hepatocellular carcinoma (HCC) and has also been confirmed as a hepatitis B virus integration site. Variants in *KMT2D* may result in abnormal enhancer regulation, which leads to changes in transcription, DNA breaks and tumor development. This also regulates hepatic metabolism and coactivates PPARy2, which leads to increased bile acid levels, which have been linked to hepatic tumor development (unspecific marker). Whether or not KS should be considered a risk factor for hepatocellular carcinoma remains unknown [93]. The association between KS and autoimmune diseases has been described in the literature (see *Immunology* section) [94]. In one case, an increased risk of Crohn’s disease development was demonstrated [95].

## 7. Otolaryngological Manifestations in Kabuki Syndrome

### 7.1. Outer and Inner Ear Features

Kabuki syndrome (KS) patients commonly present with external ear dysmorphism. In fact, this is one of the most characteristic findings of KS, present in upwards of 80% of patients [96]. Outer ear dysmorphism may include dysplasia, enlargement, external rotation, low set or a cup shape. It is present in nearly all KS patients and in many cases may support the clinical suspicion [96]. More rarely reported external ear abnormalities include microtia, preauricular fistula and pretragal pits [97]. Auditory canal atresia, dysplastic ossicles and malleus-incus fusion without stapes articulation have also been reported [97]. The internal hearing organ may also be dysmorphic, though this phenomenon is significantly rarer, present in less than 10% of patients [96]. The most common inner ear malformation in KS patients is the Mondini dysplasia: incomplete partitioning and reduced number of turns in the cochlea, enlarged vestibular aqueduct and a dilated vestibule [98]. Table 1 presents ear dysmorphism in KS patients reported in the literature.

Ear dysmorphism is often present together with clefting of the palate, lip or even the uvula. These dysmorphic traits increase the risk of otitis media in KS patients. Indeed, one of the key features of KS is recurrent acute otitis media (AOM) and the consequent necessity for recurrent antibiotic therapy. In vitro models show that recurrent AOM may be caused by disorders of eustachian tube development in vitro. This clinical picture is similar to that of other diseases characterized by craniofacial malformations [100]. Recurrent AOM puts children at increased risk for chronic complications—namely open mastoid cavity, antro-atticotomy and tympanosclerosis [96]. KS children and healthy children are treated identically for recurrent AOM [100]. These infections generally cause conductive hearing loss. KS patients with conductive hearing loss have suffered from complications of recurrent AOM, thus highlighting the importance of extensive audiological evaluation and vestibular assessment in KS patients [96]. Ear abnormalities in the context of KS may also lead to speech impediment [98]. A 9-year-old child with genetically diagnosed KS and anomalies of the hearing organ, specifically microtia, fused and malformed inner ear bones, dysplasic vestibule and posterior semicircular canals, had recurrent episodes of AOM which eventually led to hearing loss and additionally caused speech impediment [97]. This case highlights the importance of early diagnostics and the consideration of further preventative medical procedures in order to potentially decrease the severity of hearing loss and speech impediment. Six KS patients who all presented with recurrent otitis media were assessed for speech and language development. Expressive language abilities were impaired. Poor morphosyntactics (word and grammar formation), lexical and pragmatic abilities were also present, whereas strict phonological abilities were less severely affected. Phonological and morphosyntactic communication disorders represent the core problem of speech development in KS, whereas mechanical and phonological disorders only contribute to the problem additionally [101]. However, it has been suggested that speech development disorders in KS vary between individuals and are multifactorial in nature—they depend on the extent of mental retardation, hearing loss, neurological clinical picture as well as structural abnormalities of the speech organ [101].

Inner ear abnormalities (among them Mondini dysplasia) cause sensorineural hearing loss in KS patients. One KS patient manifested severe inner ear dysplasia, cochlear aplasia, semicircular canal dysplasia and unilateral vestibular enlargement. Another presented with bilaterally large ears, posteriorly rotated, underdeveloped antihelices and unilateral vestibular enlargement. Both children had profound sensorineural hearing loss, most likely due to recurrent AOM [102]. Cases of inner ear malformations without middle ear malformations (a unique clinical picture) are also known. Mondini dysplasia can be present in this situation. Hearing function depends on the changes in the stria vascularis of the spiral ligament, organ of Corti, spiral ganglion and the cochlear system. Clinically, sensorineural hearing loss as a direct result of Mondini dysplasia is expressed by high tone loss, as was seen in three cases [98]. A 7-year-old KS girl suffering from severe bilateral deafness as a result of Mondini dysplasia was successfully treated surgically. Early surgical prevention and treatment of perilymphatic fistulae in order to prevent meningitis is indicated [103].

The chronic otitis media problem has been described in the context of KS immunological issues. The majority of patients had recurrent otitis media and hearing impairment, lower levels of memory cells, severe hepatitis-B antibody deficiency (despite vaccination) and immunoglobulin deficiency. Recurrent ear infections were attributed to congenital immunodeficiency (see *Immunology* section). Notably, all patients were positive for novel *KMT2D* variants, which have been demonstrated to affect B-cell development and the regulation of signaling necessary for lymphopoiesis. The potential effects of *KMT2D* on immunoglobulin levels are unknown. Regular intravenous immunoglobulin supplementation during young ages as a treatment for immune deficiency may be indicated [104].

Therapies for otolaryngological manifestations in KS patients include initial conservative management of impaired hearing (rehabilitation and hearing aid fitting), antibiotic and symptomatic treatment of chronic otitis media, and surgical correction of the underlying disease as a last resort [105]. The bonebridge implantation procedure may aid in hearing loss treatment and thus lead to improved quality of life in KS patients. A KS boy underwent several unsuccessful surgical procedures due to mixed hearing loss associated with chronic otitis media. Ultimately, what seemed to greatly improve his quality of life was subtotal petrosectomy and an active middle ear implant—he regained social hearing skills. The active middle ear implant and subtotal petrosectomy may be effective in life quality improvement for mixed hearing loss treatment in KS [104]. Cochlear implantation, however, is not the best rehabilitation choice for all KS patients because speech therapy and hearing intervention tend to lead to better results. It may improve quality of life in patients with functional spiral ganglion cells [106].

### 7.2. Craniofacial Features

The craniofacial and dental features in the context of KS were outlined. The cardinal dysmorphism is a peculiar face with long or wide palpebral fissures, lower lateral eyelid eversion, arched eyebrows with the lateral third dispersed, prominent ears, depressed nasal tip, and skeletal and dermatoglyphic abnormalities. A typical combination of facial features is present in virtually every KS patient. Midface hypoplasia with or without mandibular hypoplasia is observed in some patients [107]. Recent mouse models tested the effect of *KMT2D* variants in neural crest cells on craniofacial dysmorphism development. Knockout mice demonstrated reduced frontonasal bone lengths and dysmorphism including cleft palate, hypoplastic mandible and cranial base ossification deficit. This was proven to be caused by altered osteochondral progenitor differentiation. Specifically, mutant neural crest cells led to defective secondary palatal shelf elevation with reduced extracellular matrix. Mutant cranial base chondrocytes do not properly differentiate and exhibit defective endochondral ossification [108,109]. Table 2 presents craniofacial dysmorphism in KS.

## 8. Orodental Symptomatology

Oral anomalies are present in over 60% of KS cases. Documented orodental pathology cases include congenital tooth absence, malocclusion, high-arched palate, abnormal dentition, widely spaced teeth, hypodontia, conical incisors, screwdriver-shaped incisors, delayed tooth eruption and ectopic upper molars [113,115,116]. The role of *KMT2D* and *KDM6A* genes in tooth development has been recently theorized. Currently, the maxillomandibular relationship, tooth size and dental arch forms are being studied. The expression of *KMT2D* and *KDM6A* in human tooth buds at 7–12 Hbd was recently demonstrated. Gene expression was observed in the dental epithelium of primary incisors, canines and molars at the bud and cap stages of tooth development. This suggests that loss-of-function *KMT2D* or *KDM6A* variants may underlie the observed orodental anomalies [113]. The function of *KMT2D* and *KDM6A* as epigenetic modulators of several biologic processes was recently discussed. Loss of their function leads to a variety of manifestations, in this context namely hypodontia, widely spaced teeth, absence of teeth, enamel hypoplasia, dental agenesis and caries [116]. Cephalometric analysis can give insight to craniofacial structure and development. Using cephalometry, they found that severe maxillary recession and midfacial hypoplasia are common among KS patients. This is practical for the clinical diagnosis of KS [115]. Cephalometric follow up of one patient showed the improvement of maxillary and mandibular growth in their patient. During this period, proper dental care, tooth extractions and fluoride treatments were ordered [114]. Tooth buds for lateral upper and lower incisors as well as molars may be absent [110]. Incisors may have large pulp chambers, may be affected by external root resorption or root canal division, tooth retention, retrognathia of the upper jaw, clefting, or lower lip fistula [110]. KS cases with taurodontism (a condition in which the molar body and pulp chamber is enlarged at the expense of the roots, leading to apical displacement of the tooth furcation) were described [110,117]. These dental findings may help support the clinical diagnosis of KS [117]. Tooth extractions and fluoride treatment seem to be effective treatment methods. Missing permanent teeth, sometimes up to seven in total, can also be confirmed in KS patients. This is most commonly true of maxillary and mandibular incisors; however, a missing maxillary canine can also be seen [119]. Nursing bottle syndrome likely caused screwdriver-shaped incisors and high-arched maxilla manifested poor oral hygiene and early childhood caries in one KS boy, which highlights the importance of dental health in the improvement of life quality. KS patients may be more prone to root resorption than healthy patients due to their organic disease. Thus, their orthodontic state must be monitored [120]. 

The treatment of orodental symptoms includes orthodontic treatment to correct bite function in addition to esthetics. Extraction and implantation procedures are often used to correct disorders of tooth development and increase patient quality of life. Orthodontic treatment can also be aimed at correcting arch length discrepancy. Due to feeding difficulties and an increased risk of nursing bottle syndrome, dental and orthodontic care must be integrated with child speech therapy in order to maintain long-term effects of orthodontic treatment. This aims at quality of life improvement [101,118,120].

Cleft palate is a feature observed in 33–50% of KS cases. Pediatric follow-up is crucial in KS patients with clefting. Usually the first symptoms of cleft palate are speech development disorders and nasal leakage of milk during early infancy (submucous cleft palate). They mention, however, that mental retardation often complicates the accurate correct diagnosis of cleft palate because the above symptoms may be attributed to it. Therefore, they suggest that cleft palates are more frequently present than reported. They highlight the importance of routine specialist examination for clefting [112]. KS children with cleft palate suffered from velopharyngeal insufficiency and ended up requiring two-flat palatoplasty procedures. These interventions ultimately improved speech development and quality of life [111]. Another KS children cohort with cleft palate also had drooping lower lips, which is a symptom that may aid in the clinical diagnosis of KS [121]. A KS girl whose parents are first cousins presented with multiple congenital abnormalities, including anorectal malformation—specifically imperforate anus with recto-vestibular fistula—diaphragmatic defect, lower lip pits, hypopigmentation, hypogammaglobulinemia as well as cleft palate. The severe concomitantly present multiple congenital abnormalities are likely to worsen prognosis. No other KS patient was reported with such severe anomalies [122].

Lower lip pits are another feature that has been reported in KS, albeit more rarely than other cardinal features. Two KS patients were initially diagnosed with Van der Woude syndrome (a condition that affects facial development—lower lip pits are a cardinal feature of said syndrome, often with some combination of cleft lip and palate). These patients presented with lower lip pits and cleft palate and were clinically diagnosed with Van der Woude syndrome in early infancy. Upon follow-up during the second to fourth years of life, it was consequently noticed that they exhibited clinical features that do not concur with Van der Woude syndrome: facial dysmorphism—widened palpebral fissures, eyelid eversion and prominent ear; chronic otitis media leading to hearing impairment; velopharyngeal insufficiency and delayed psychomotor development. These patients’ clinical picture resembled KS, which was later confirmed by genetic testing. These cases emphasize the need for the careful evaluation of patients with lip pits and raise awareness for differential diagnosis [99].

## 9. Ophthalmologic Issues in Kabuki Syndrome

**Figure 1:**
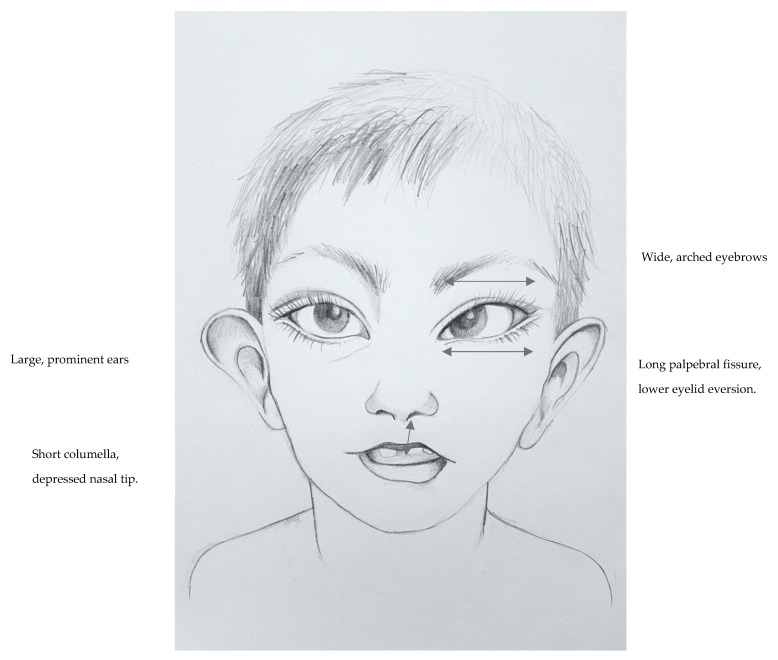
A child exhibiting cardinal Kabuki syndrome facial dysmorphism: long palpebral fissures with eversion of the lateral third of the lower eyelid; wide, arched eyebrows with sparseness or lack of the lateral third; short columella (lower part of the nasal septum) with depressed nasal tip; large, prominent or cup-shaped ears; persistent fetal finger pads.

Extraocular features have been defined as cardinal clinical diagnostic signs of Kabuki syndrome. They are present in nearly all KS patients [123] and include: long palpebral fissures, lower palpebral eversion, arched eyebrows, epicanthus, ptosis, and a Marcus Gunn pupil (a type of relative afferent pupil defect that causes a pathologically decreased pupillary light reflex). These and other cardinal KS dysmorphisms are presented in Figure 1. Another frequent feature occurring in over a half of KS patients is nocturnal lagophthalmos (sleeping with open eyes). It can predispose patients to infectious complications due to dry eye [123,124]. Ocular signs comprise amblyopia (“lazy eye”), refractive error, strabismus, nystagmus, microphthalmia, retinal or disc coloboma, and optic disc anomalies [123,125,126]. Strabismus may take the form of alternate convergence as well as esotropia, including large-angle congenital esotropia [126,127]. Caruncular lipoma, bilateral blepharitis (most likely as a complication of nocturnal lagophthalmos) and bilateral inferior corneal pannus (fibrovascular granulation tissue) have been described in single patients [124]. Ocular abnormalities often affect visual function. The prevalence of amblyopia and impaired acuity may be underestimated because many patients with intellectual disability do not undergo visual acuity tests. Vision disorders tend to worsen development; therefore, careful ophthalmologic evaluation at as young an age as possible is critical for each patient in order to correct treatable visual impairments [123].

Coloboma is frequently reported in KS patients. It is sometimes associated with congenital heart disease and ear and renal defects, leading to the misdiagnosis of CHARGE syndrome before the presentation of typical facial dysmorphisms. It may affect the iris, choroid, retina or optic nerve. Coloboma derives from incomplete embryonic fissure closure in utero. Normally, the embryonic fissure develops from an invagination of the optic vesicle and leaves a gap that lets the hyaloid artery supply the inner eye, a process crucial for normal development. Iris or ciliary body colobomas result from incomplete anterior closure, choroid, retina or optic nerve colobomas result from incomplete posterior closure, whereas lens colobomas may be caused by incomplete closure of any segment. Eyelid colobomas are of a different embryologic origin and arise at a later stage of development. These are generally unrelated to disorders of globe development [128]. CHARGE syndrome may phenotypically resemble Kabuki syndrome [129]. It is genotypically different from KS (variants in *CHD7*—a gene coding for a helicase) [130]. Infrequent features that are shared between KS and CHARGE include microphthalmia, coloboma, anal atresia and panhypopituitarism. This similarity may be explained by the modifier genes effect [130].

A wide array of rare ophthalmologic findings is present in almost all Kabuki syndrome individuals. Currently, it is not possible to directly associate these features with KS, yet a recommendation is widely supported that all KS patients should undergo ophthalmological assessment at the earliest age possible [125]. Abnormal corneal development in utero has been demonstrated to lead to congenital corneal staphyloma—a severe corneal defect that causes a forward projection between the eyelids. The anomaly is now linked with *KMT2D* variant [131]. Bilateral congenital corneal opacity as an early-onset ocular KS manifestation has also been described. This is a rare congenital loss of corneal tissue transparency originating between the 6 and 16th week of gestation. In the same patient, bilateral corneal transplantations were performed in order to prevent deprivation amblyopia [132,133]. Agenesis of the lacrimal punctae was found as an incidental finding in a 29-year-old patient with *KMT2D* variant [134]. A KS patient with typical periorbital dysmorphism (lower eyelid eversion, epicanthus inversus, depressed nasal root, high-arched eyebrows, prominent ears and prominent eyelashes) underwent a simultaneous surgical repair of medial and lateral canthus. The deformity was characterized by raphe dysplasia in the orbicularis oculi muscle, which was corrected satisfactorily by lateral tarsorrhaphy—a partial surgical closure of the lateral part of the eyelids. In this case, surgical correction was recommended to prevent the drying of the cornea and conjunctiva, besides esthetic considerations. The surgical correction of canthus deformity should be considered in KS patients in whom corneal dryness may affect eye function [135].

Microphthalmia was described in several KS patients. In one neonate, extreme microphthalmia with an anomaly of globe development, hyperplastic primary vitreous and hypoplastic optic chiasm were all present [136]. *KMT2D* variant was later confirmed in this patient. *KMT2D* proteins are active as part of a multi-subunit complex (see *Molecular Genetics* chapter) and have been known to interact with *CHD7* and *CHD8* proteins through the WAR complex. Additionally, *KMT2D* has been known to interact with the *PAX*-interacting protein 1 (*PAXIP-1*) as well as other transcription factors encoded by *PAX2*. These factors have a high rate of expression in the developing eye, which may also explain microphthalmia and eye developmental disorder in utero [136]. Ocular muscle dysfunction was recently reported. The overaction of inferior oblique muscles together with weakness of the superior oblique muscles associated with a V-shape motion was observed in four patients, and the underestimation of this phenomenon in KS patients has been put forward.

A summary of other rare ophthalmologic findings that were reported in under 10 KS patients in the literature is presented in Table 3 below. Many of these ophthalmologic findings tended to affect the cornea.

## 10. Oncological Issues in Kabuki Syndrome

*KMT2D* is expressed in most cells and tissues. Its pathogenic variant results in the interruption of histone methylation related to gene expression, thus affecting normal growth and development as well as many other processes described in other chapters. Its role in development, metabolism, cell differentiation and tumor suppression has been recently described [145]. Defective histone demethylation, as a result of a somatic variant, may dysregulate gene expression and predispose to cancer. *KMT2D* has been defined as one of the most commonly mutated genes in a number of cancers such as gastric cancer, lymphoma and medulloblastoma [145]. The lung-specific loss of *KMT2D* promotes lung tumorigenesis and upregulates tumorigenic processes such as glycolysis in a recent mouse model [146]. The oncogenic potential of *KMT2D* loss-of-function variant has also been demonstrated; however, the rarity of such variants makes it difficult to assess their pathogenicity exactly [147]. Using recombination and nuclease-mediated gene editing, it was proven that a *KMT2D* loss-of-function variant causes the proliferation of neoplastic cells [148]. This pathomechanism is likely responsible for medulloblastoma formation [149]. Additionally, *KMT2D* deficiency attenuates cancer cell migration, promoting tumor growth [148]. The study also confirmed previously published findings which showed that *KMT2D* is required for effective H3K4 methylation [148].

*KMT2D* and *KDM6A* belong to the same beta-globin and estrogen-receptor regulator multiprotein complex (called ASCOM) and interact with each other [145,150]. Recently, *KDM6A* has been shown to interact with CBP transcription-activator protein in a breast cancer cell-line *MCF-7* in a *Drosophila* model. Clinically, this variant has been found in many tumor types, including multiple myeloma, esophageal squamous cell carcinoma, renal cell carcinoma, glioblastoma, and breast, urinary tract, pancreatic and colorectal cancers. *KDM6A* has been demonstrated as a tumor suppressor that leads to slow cell growth [151,152,153]. Several mouse models analyzed *KDM6A* in the context of urinary tract, pancreatic and lung tumors and acute myeloid leukemia [151]. Conversely, *KDM6A* has a pro-oncogenic role. The loss of *KDM6A* in human breast cancer cells causes a decrease in estrogen-induced cell proliferation in vitro. This is, however, based on one recent study and conclusions as for in vitro pro-oncogenic trends of *KDM6A* are yet to be determined [151].

The above described role of somatic *KMT2D* variants, i.e., restricted to cancer tissue, is well known. However, the significance of germline (present in all tissues) pathogenic variants in this gene in KS patients is yet to be determined. KS patients may present with a wide range of tumors. The new research on growth hormone therapy (see *Growth* chapter) suggests that it may worsen tumor burden [145]. Cancer risks, on an individual basis, should be established in KS patients in whom growth hormone therapy is considered.

In a 3-year-old KS patient in whom Wilms tumor was diagnosed, no negative impact on lifespan was demonstrated and the tumor was successfully managed according to procedures used in non-KS patients [154]. Neuroblastoma, the most common extracranial solid tumor in infancy, was described in clinically diagnosed KS patients. This patient’s prognosis was assessed to not be different from sporadic cases, but constitutional molecular defects associated with KS may play a role in oncogenesis [155,156]. The tumor spectrum was expanded to include: a low-grade fibromyxoid sarcoma in a clinically diagnosed KS patient—the mass was successfully resected [157]; and a case of aggressive desmoid fibromatosis, successfully treated surgically [158].

Hepatoblastoma was described in one 6-year-old clinically diagnosed KS patient. Prognosis was assessed to be favorable and this patient was treated with chemotherapy (which led to mild hematotoxicity). Later, a right lobe hepatectomy was performed, followed by adjuvant chemotherapy, successfully leading to complete remission [155]. Hepatocellular carcinoma was reported in another KS patient, a 15-year-old female with a *KMT2D* variant. She underwent donor liver transplantation. On biopsy, besides characteristics of hepatocellular carcinoma, the explanted liver exhibited hepatic adenomas with a low Ki-67 proliferation index [93]. The association between genetic syndromes and liver cancer has been suggested [93,159]. *KMT2D* has been shown to be mutated in hepatocellular carcinoma cases, and has even been suggested to be associated with earlier recurrence of disease, greater microvascular invasion and a more aggressive phenotype [93,160]. It has also been suggested to disrupt the control of bile acid production in mutant mice [93,161]. Despite the cases above presenting a benign phenotype, the potential development of malignant liver cancer must be considered in KS patients.

Pilomatricoma—a benign hair cell tumor characterized by lobules of epithelial cells—has been associated with several genetic syndromes (Turner, Gardner, Rubinstein-Taybi, Sotos, Trisomy 9, myotonic dystrophy, spina bifida, sarcoidosis, gliomatosis cerebri) [162,163,164]. The only known tumor that has been confirmed in pilomatricoma is caused by a somatic variant in *CTNNB1*. KS may predispose patients to pilomatricoma formation because *CTNNB1* plays a role in the *WNT* pathway, which is regulated by *KMT2D* [162,163]. Surgical excision is indicated [164]. Another dermatologic lesion, Becker nevus, was also reported in KS [165].

Giant cell fibroblastoma, a soft-tissue malignancy that is especially rare in childhood, was reported in a 12-year-old female patient with a *KMT2D* variant. She presented with a recurring neck mass despite excision and received a favorable prognosis. The tumor was successfully excised [166].

Spinal ependymoma was diagnosed in a 23-year-old KS patient. Ependymoma is a tumor originating from the ependymal lining of the ventricular system. Most cases are located intracranially; however, spinal presentation is less common. The patient presented with hypoesthesia of the feet and lumbar pain. Laminotomy tumor resection was performed and no neurological complications were observed. Ki-67 index was low upon biopsy. This case sheds light on atypical symptom presentation in KS patients—even though ependymoma does not typically present in the spine, spinal abnormalities are typical in KS patients [167].

Several KS patients with hematological malignancies were reported. Hodgkin Lymphoma was reported in a patient with a severe multiorgan phenotype and *KMT2D* germline variant. He presented with clubfeet, hirsutism, hypotonia, and feeding problems during infanthood; chronic otitis media, obesity and failure to thrive during late childhood; and confirmed Immunoglobulin IgA and IgG deficiencyType 1 diabetes mellitus1 and pancreatic failure in young adulthood. He required pancreatic transplant thrice, after which he developed Ebstein-Barr virus-positive Hodgkin Lymphoma and underwent chemotherapy, achieving complete remission. *KMT2D* is one of the most frequently mutated genes in follicular lymphomas as well as in diffuse large B-cell lymphomas. By the loss of H3K4 methylation at enhancer DNA regions, truncating variants of the *KMT2D* protein lead to the loss of enzymatic activity and haploinsufficiency, causing malignant transformation [168,169]. A clinically diagnosed KS patient with IgA and IgG deficiency developed acute lymphocytic leukemia. She had a history of recurrent infections, indicating an immunodeficiency with susceptibility to cancer [170]. Several cases of KS patients with Burkitt lymphoma were also reported [171,172,173,174]. In the most recently reported case, genetic testing was available to reveal a *KMT2D* variant causing KS as well as a translocation t (8;14) causing Burkitt lymphoma in an EBV-positive KS patient. KS patients may thus be at increased risk for hematological cancers. Decreased histone regulation may cause DNA damage due to *KMT2D* variant, and that may increase the risk for other permanent DNA damage to occur, in this case, t (8;14) translocation [174]. Table 4 describes rarely reported tumors in KS patients.

## 11. Immunological Issues in Kabuki Syndrome

KS patients present with numerous immunopathological manifestations. *KMT2D* and *KDM6A* encode, among others, proteins acting in the COMPASS complex, which serves as a chromatin opener. Loss of function of *KMT2D* and *KDM6A* epigenetically leads to defective B-lymphocyte terminal differentiation and thus hypogammaglobulinemia and reduced memory B-cell numbers [174]. B-cell lineage development deficiencies were demonstrated in a recent mouse model. Mice with a *KMT2D* variant had reduced serum IgA and elevated IgM. The bone marrow, spleen and intestine of *KMT2D*-mutated mice contained fewer IgA-secreting cells. There were more B-lineage cells in the Peyer patches; however, there were fewer Peyer patches overall. This was also found in vivo. *KMT2D* epigenetically controls the gene *ITGB7*, which encodes for an adhesion protein that mediates intestinal homing (the process by which white blood cells target both inflamed and non-inflamed regions of the gut to provide an immune response). *KMT2D* insufficiency has deleterious effects on B-cell differentiation, explaining this mechanism of immunodeficiency in KS patients [175].

*KMT2D* takes part in chromosomal translocations that create chimeric proteins. Translocation partners exist in a super elongation complex, which is required for *Hox* gene expression in white blood cells as well as in leukemic cells (potentially increasing the risk of leukemogenesis, see *Oncology* chapter) [150]. The COMPASS complex epigenetically regulates FOXP3, which affects the differentiation of naïve CD4+ T-cells into T-regulatory cells. Reduced T-cell tolerance also explains the increased risk of autoimmune pathology observed in KS patients.

Considering the genotype, the prevalence in KS patients of immune deficiency or autoimmune manifestations does not differ by gene type (whether *KMT2D* or *KDM6A*). An increased risk of immune thrombocytopenic purpura development with missense variants than with truncating variants has been suggested [176]. The immunological phenotype observed in KS patients has been recently described in a large cohort and includes (in order of prevalence) infection susceptibility (especially to acute otitis media—see *Otolaryngology* chapter), hypogammaglobulinemia, increased risk of autoimmune disease, increased risk of immune thrombocytopenic purpura, vitiligo, autoimmune hemolytic anemia, thyroiditis and other rare autoimmune cases [176]. The prevalence of immune thrombocytopenic purpura usually decreases with age in healthy children; conversely, its prevalence slightly increases in KS patients [176]. KS patients have an increased risk of developing refractory immune thrombocytopenic purpura, for which Rituximab seems to be an effective treatment method [177]. IVIG and steroid treatment of immune thrombocytopenic purpura and autoimmune hemolytic anemia seems to be equally effective as in healthy children [178,179].

KS patients are more likely to develop Common Variable Immunodeficiency, which is known to increase the risk of autoimmune disease development, especially immune thrombocytopenic purpura and autoimmune hemolytic anemia [180]. The KS immunological phenotype has been compared to that of common variable immunodeficiency, which is characterized by recurrent bacterial infections, hypogammaglobulinemia and impaired antibody responses as well as an increased prevalence of inflammatory and autoimmune disorders [181,182,183]. There is heterogeneity of immune pathology in KS [182]. Decreased or even undetectable IgA levels are observed in over 80% of KS patients. Low IgG levels are seen in nearly half of KS patients [174,184]. It is recommended that each KS patient be regularly tested as blood counts, IgA levels and urinalysis may present early clues for immune abnormalities. Patients with recurrent otitis and low levels of serum Ig should be consulted by an immunologist (see *Otolaryngology* chapter). The routine administration of IVIG has not been proven to be effective in every KS patient. IVIG should only be considered in case of low total serum IgA [182,183]. *MTOR* inhibitors, as a more targeted treatment for immunodeficiencies associated with KS, are a subject of recent research, and singular case studies present the remission of cytopenia (see Table 6) [184].

Table 5 and Table 6 present singular KS cases of autoimmune and immunodeficiency manifestations, respectively, reported in the literature. These cases suggest a broadened KS immunological phenotype.

## 12. Nephrological Issues in Kabuki Syndrome

Up to 40% of KS patients present with urinary system abnormalities. Of these, about half present with renal malformations and half with urinary tract anomalies. The concomitant presentation of renal and urinary tract abnormalities is rare. Renal malformations and diseases include, in order of prevalence, horseshoe kidney, renal hypodysplasia, renal ectopy and renal duplication and renal insufficiency (single reported cases). Urinary tract malformations include, in order of prevalence, hydronephrosis and ureteral duplication. No phenotype correlation between KS and vesicoureteral reflux or ureteropelvic junction obstruction seems to be prevalent [189]. Considering genotype-phenotype correlation, it is suggested that *KMT2D* variant increases the risk of renal manifestations, but not of urinary tract anomalies [1,189,190]. The etiology of urinary system abnormalities in the context of KS has been suggested to be rooted in the *KMT2D* epigenetic role in the regulation of renal development. Dysfunctional *KMT2D* may ultimately lead to renal dysplasia. However, the specific role of *KMT2D* in renal development has not been established [190].

Several KS patients with severe renal insufficiency have been reported. Some of these patients also presented with renal dysplasia. One KS patient presented with severe renal insufficiency during the first month of life due to dysplastic kidneys. She had a positive family history of Polycystic Kidney Disease. Poor prognosis was concluded [189]. Another KS patient presented with terminal renal insufficiency due to bilateral renal dysplasia at the age of 6 years. She underwent successful transplantation and prognosis was good [191]. Another KS patient with a nonfunctional dysplastic kidney underwent successful transplantation at the age of 14 [192]. One KS patient did not undergo renal transplantation in time and died at the age of 5 years due to renal failure and concomitant pulmonary hypertension [26]. Renal hypoplasia characteristics can be seen upon renal ultrasonography and may be associated with increased risk of renal insufficiency. This suggests that regular ultrasonographic assessment, blood pressure measurement, glomerular filtration rate evaluation and proteinuria screening are necessary as preventative measures in KS patients. If worsening kidney function is suspected, nephrotoxic drugs and high-protein diet should be avoided. These patients should be promptly referred to a pediatric nephrologist as they can often be treated with good prognosis [189].

Kabuki syndrome has often been compared to Au Kline syndrome while considering kidney diseases. Au Kline Syndrome is caused by a variant in *HNRNPK*, which codes for a ribonucleoprotein. The variant causes a multiorgan development disorder among other effects, ultimately leading to chronic kidney disease and kidney failure. Renal anomalies leading to chronic kidney disease are not unheard of in KS patients too. Renal ultrasound, kidney function evaluation and, when appropriate, early dialysis are recommended in both Au Kline Syndrome and KS patients too [193].

Generally, the treatment of renal anomalies in KS patients is no different than in patients without KS [189]. Several successful renal outcomes of kidney transplantation in KS patients have been reported in the literature [192,194]. Considering the progressive nature of the urinary system malformations, renal transplantation may be a realistic treatment option for KS patients (see Table 7).

Rare urinary system manifestations reported in the literature are presented in Table 7 below. These cases often present with concomitant clinical findings, namely congenital heart defects and ear disease (recurrent acute otitis media and/or ear malformation).

## 13. Conclusions

It is critical that general practitioners are aware of KS. Dysmorphic features in KS patients may appear similar; however, the entire clinical picture may vary widely between patients. This is especially true considering issues pertaining to specific organ systems, as we have demonstrated in our review. Early genetic diagnostics to detect the underlying syndrome are crucial as they confirm KS as well as classify the KS subtype. The early recognition of abnormal medical issues in KS patients is important to prevent complications and to improve prognosis, especially considering that the treatment of certain conditions in KS patients is no different in patients without KS. A greater understanding of the clinical presentation of KS will allow doctors to provide quality primary care to patients and to their families.

Further research should focus on the specific genetic pathomechanism of the various clinical states we describe in order to potentially develop novel treatments. Mouse models and computer models are being studied. Existing treatments (for example, growth hormone supplementation) should be another subject of further research. Large cohorts of KS patients should be recruited for a longitudinal study as current findings remain limited. Finally, an in-depth analysis of KS patients’ quality of life should be performed in order to shed light on the most helpful quality of life improvement techniques as well as, we hope, to prepare for the coming age of genetic therapy.

## Figures and Tables

**Table 1:** Ear dysmorphism in Kabuki syndrome (KS) patients reported in the literature.

Characteristics	Frequency (%)	Cases	Citations
Outer ear dysmorphism: dysplasia, prominence, external rotation, low-set, cup shape	80–100	4	[96]
Microtia		1	[97]
Hearing loss	24–65		[96,98,99,100]
Chronic otitis media	55–90		[96,100]
Inner ear abnormality: Mondini Dysplasia		3	[98]

**Table 2:** Craniofacial dysmorphism in KS patients reported in the literature.

*Characteristics*	Frequency (%)	Cases in Literature	Citations
*Soft palate paralysis*		3	[98]
*Cleft palate (general)*	35–70	15	[98,99,102,110.^111^,^112^,^113^]
*Submucous cleft palate*	15–50		[111]
*Lower lip pits*	10	4	[99]
*Lower lip nodules*	73		[99]
*Characteristic facies*	100		[99,110,114,115,116]
*Eyelid eversion*	87–100	21	[99,107,110,113,116]
*Depressed nose*	80–100	20	[99,107,110,113,116]
*Arched eyebrows*	68–90	23	[107,110,113,115,116]
*Epicanthus*	60–65	4	[115,116]
*Low posterior hair line*	50–55	5	[115,116]
*Strabismus*	45–50	5	[115,116]
*Preauricular dimple*	39	5	[115]
*Abnormal dentition*	48–85	20	[99,110,113,114,116,117]
*Tooth absence, malocclusion*	80–90	9	[107,113,117,118]
*High-arched palate*		7	[113]
*Bifid uvula*		1	[112]

**Table 3:** Rarely reported significant ocular findings in KS patients. ADHD—Attention deficit and hyperactivity disorder AOM—acute otitis media; ASD—atrial septal defect; IgG—Immunoglobulin-G; VSD—Ventricular septal defect; PDA—patent ductus arteriosus, SVC—superior vena cava; UTI—urinary tract infection.

Eye Organ	Rare Ocular Findings	Concomitant Ocular Findings	No. Reported Pts	Pts Age	Genetic Variant	Other Clinical Features	Citation
Caruncle	Lipoma	None	1	3 months	Unknown	Developmental delay, failure to thrive, KS facial dysmorphism, brachycephaly, low-set ears, micrognathia, single palmar crease	[124]
Eyelid	Blepharitis				Unknown		
	Jaw-winking ptosis	Astigmatic amblyopia, wide palpebral fissures, anisocoria, sparse eyebrows	1	7 years	Unknown	Not mentioned	[137]
Cornea	Pannus				Unknown		[124]
	Staphyloma (with and without microphthalmia)	Typical KS periocular dysmorphism	2	1 month	*KMT2D*	(1) Low-set ears, arched palate, sacral dimple, VSD, double-renal pelvis, hydronephrosis, ureteropelvic junction stenosis, renal malrotation, psychomotor developmental delay. (2) VSD, ASD, heart failure, epilepsy, global developmental delay	[131]
	Opacities	None	1	At birth	*KMT2D*	Short 5th finger, PDA, absent uvula, ectopic kidney	[132]
	Salzmann Nodular Degeneration	Nystagmus, cataract, decreased acuity, arched eyebrows, lower eyelid eversion, prominent eyelashes, long palpebral fissures	1	18 years	None—Clinical diagnosis only	Typical KS dysmorphism, VSD, hypospadias, cryptorchidism, single umbilical artery	[138]
Disc	Dysplasia	Ptosis; esotropia; coloboma of iris, retina, choroid, optic disc; microcornea	1	3 years	*KMT2D*	Spinal dysraphism, short stature, intellectual disability, typical KS facial features,	[139]
Globe	Microphthalmia (with coloboma)						
	Congenital refractory glaucoma	Bilateral trabeculectomy, right retinal detachment with phthisis bulbi	1	3 months	Not mentioned	Orofacial cleft, depressed nasal tip, micrognathia, arched eyebrows, low-set ears, short stature, low body weight, brachydactyly, developmental delay	[140]
Lacrimal puncta	Agenesis	Bilateral long palpebral fissures, eversion of lower eyelid, prominent eyelashes, broad eyebrows with lateral notches, decreased visual acuity, retinal coloboma, blepharoptosis	1	29 years	*KMT2D*	Epilepsy, neurosensory deafness, double aortic arch, mitral dysplasia, bivalve aorta, left SVC draining coronary sinus, mental retardation, typical KS facial dysmorphism	[134]
Retina	Tortuous vessels, peripapillary gliosis	Refractive error, strabismus, retinal pigmentation, arched eyebrows, prominent eyelashes, lower eyelid eversion, long palpebral fissures	1	21 years	Unknown	Malformed ears, brachydactyly, fetal fingertip pads, intellectual disability, short stature	[141]
	Macular degeneration	None	1	9 years	Unknown	Failure to thrive, gynecomastia, typical KS dysmorphism, short 5th phalanges	[142]
	Macular deposits	Decreased visual acuity	1	6 years	Unknown	Recurrent AOM, speech delay, typical KS dysmorphism	[143]
Periocular muscles	Overaction of inferior oblique, weakness of superior oblique	Strabismus with esotropia, astigmatism, ptosis	4	2–10 years	*KMT2D*	(1) Immune thrombocytopenia, trigonocephaly. (2) velopalatine insufficiency, hypotonia, frequent UTI, IgG hypogammaglobulinemia, intellectual disability, hypovitaminosis D, iron deficiency anemia, obesity, precocious puberty, trichotillomania, factor 7 deficiency. (3) chronic rhinitis, VSD, recurrent bronchiolitis. (4) ADHD	[144]

**Table 4:** Rarely reported tumors in KS patients. AOM—Acute Otitis Media; KS—Kabuki syndrome; L—left; R—right; Tx—transplant; EBV—Ebstein–Barr Virus.

*Tumor*	Concomitant Findings	Age (Yr)	Genotype	Citation
*Fibromyxoid sarcoma*	Feeding difficulty at early age, typical KS appearance, obesity, solid mass below the R scapula	11	Unknown	[157]
*Acute Lymphocytic Leukemia*	Growth retardation, recurrent AOM, typical KS appearance, duplication of L kidney, capillary hemangioma on the back	1	Unknown	[170]
*Desmoid-Type Fibromatosis*	Developmental delay, typical KS appearance, Chiari I malformation	10	*KMT2D*	[158]
*Tanycytic Spinal Ependymoma*	Typical KS appearance, acute lumbar pain, tactile hyposthenia of the feet, lumbar endocanalar mass, successful surgical resection	23	*KMT2D*	[167]
*Giant Cell Fibroblastoma*	At an early age: breathing difficulty, urinary tract abnormalities, intellectual disability, typical KS appearance. Mass on the R side of neck.	12	*KMT2D*	[166]
*Hodgkin Lymphoma*	Typical KS appearance, unusually severe KS multisystem phenotype, immune deficiency, autoimmune pancreatitis (status post 3 pancreatic Tx).	34	*KMT2D*	[170]
*Burkitt Lymphoma*	Typical KS appearance, EBV (+), malignant abdominal tumor.	3	Unknown	[173]
*Burkitt Lymphoma*	Typical KS phenotype, urinary tract abnormalities, Burkitt lymphoma, t (8;14) translocation with *c-MYC* gene involvement	5	*KMT2D*	[174]
*Becker Nevus*	Typical KS phenotype, hyperpigmented macule on right buttock	6	Unknown	[165]

**Table 5:** Rare autoimmune manifestations reported in the literature. APLA—Antiphospholipid Antibody; AOM—Acute Otitis Media; AIHA—Autoimmune Hemolytic Anemia; ASA—Acetylsalicylic Acid; CP—Cyclophosphamide; GCS—Glucocorticosteroids; G-CSF—Granulocyte Colony Stimulating Factor; IVIG—Intravenous Immunoglobulins; MMF—Mycophenolate Mofetil, WES – Whole Exome Sequencing.

Autoimmune Disease	Concomitant Clinical Features	Genotype	Age	Treatment	Follow-Up	Reference
Systemic Lupus Erythematosus	APLA (+); persistent hypogammaglobulinemia; convulsions (1x), recurrent sepsis, typical KS appearance, brachydactyly (toe 2 bilaterally)	*KMT2D* (rare variant, WES)	17	GCS, CP, MMF, IVIG	Immunosuppression continued to date	[185]
Autoimmune neutropenia	Typical KS facies, skeletal defects, recurrent AOM; AIHA; autoimmune thrombocytopenia	*KMT2D*	6	IVIG, G-CSF	Remission to date	[186]
APLA Syndrome	Chorea (limbs); characteristic KS facies, bilateral dysmetria, low IgA	Unknown	16	ASA, Physical therapy	Symptoms gradually improved, serum APLA persistently elevated	[64]
Lymphoid Interstitial Pneumonia (nonneoplastic, inflammatory pulmonary reaction associated with autoimmune diseases, most often Sjogren syndrome)	Characteristic KS facies, hypogammaglobulinemia IgG, IgA and IgM	Unknown	18	GCS, Rituximab	Lungs cleared up soon after GCS administration; however, lung function is obstructive and restrictive, prognosis is poor	[187]

**Table 6:** Rare autoimmune manifestations reported in the literature. AOM—Acute Otitis Media; DM1—Diabetes Mellitus Type 1; FUO—Fever of Unknown Origin; GERD—Gastroesophageal Reflux Disease; ITP—Immune Thrombocytopenic Purpura; MMF—Mycophenolate Mofetil; SCIg—Subcutaneous Immunoglobulins; SFx—Side Effects; URTI—Upper Respiratory Tract Infection; VSD—Ventricular Septal Defect.

Immunodeficiency	Concomitant Clinical Features	Genotype	Age (Year)	Treatment	Follow-Up	Reference
Granulomatous Lymphocytic Lung Disease	DM1, typical KS facies, recurrent AOM	Unknown	16	Sirolimus monotherapy	Complete remission	[184]
Cerebral Lymphoproliferative Disorder	ITP, lack of typical KS facies, other dysmorphism: fingertip pads, VSD, pilomatrixoma	*KMT2D*	9	Sirolimus, MMF	Sirolimus was discontinued due to SFx; remission achieved after MMF use	[63]
Mild Humoral Immunodeficiency (IgG decreased after age 6)	Typical KS facies, recurrent URTI, recurrent AOM (treated with tympanostomy), recurrent pneumonia, recurrent FUO (infections 1x/mo). GERD (treated with Nissen fundoplication and gastrostomy).	*KDM6A*	6	SCIg	Significant improvement	[188]

**Table 7:** Rare Urinary System manifestations in KS patients. AOM—Acute Otitis Media. ARB—Angiotensin Receptor Blocker. CoA—Coarctation of Aorta. CHD – Congenital Heart Disease.DM1—Diabetes Mellitus Type 1. GH—Growth Hormone. GN—Glomerulonephritis. GCS—Glucocorticosteroids. PDA—Patent Ductus Arteriosus. RF—Renal Failure. RTx—Renal Transplantation. SFx—Side effects. VSD—Ventricular Septal Defect.

Urinary System Manifestation	Concomitant Clinical Features	Genotype	Age (yr)	Treatment	Follow-Up	Reference
Refluxing megaureter; dysplastic and nonfunctioning kidney; Stage 5 RF;	Typical KS facies, mental retardation, hearing loss, spina bifida occulta, CoA, diaphragmatic hernia	Unknown	10	Reflux: endoscopy. RF: RTx, subsequent immunosuppression	Good compliance, proper kidney function, DM1 as SFx of tacrolimus	[192]
Spontaneous infection of atrophic ureter; ectopic ureteral opening; status post RTx	Typical KS facies, developmental delay	*KMT2D*	24	Laparoscopic ureterectomy	No complication	[194]
Renal agenesis	Typical KS facies, hypoglycemia, thrombocytopenia, jaundice, CoA	Unknown	7	Symptomatic, rehabilitation	Unknown	[195]
Renal hypoplasia	Typical KS facies, VSD, bicuspid aorta with insufficiency, chronic AOM, chronic bronchitis, Rh-GH	*KMT2D*	8	Symptomatic, rehabilitation	Unknown	[195]
Non-functioning kidney	Consanguineous parents, typical KS facies, microcephaly, chalazion, enlargement of glans penis, mental retardation	Unknown	3.5	Symptomatic, rehabilitation	Unknown	[196]
Hypoplastic kidney	Typical KS facies, cleft lip and palate, PDA, VSD, CoA, poor ear formation-clinical picture suggestive of both KS and Malpuech syndrome	Unknown	9.5	Surgical correction of CHD and clefting	Positive effect	[197]
Renal failure	Typical KS facies, developmental delay, ataxia, IgA deficiency, Hashimoto Thyroiditis, Vitiligo, CHD (ASD, pulmonary stenosis),	Unknown	6	Renal transplant	Positive effect	[191]
Hepatorenal failure	Typical KS facies, webbed neck, postnatal growth retardation, frequent infections (suspicion of immunocompromization)	Unknown	0.5	Liver transplant	Positive effect, recurrent UTI	[191]
Renal failure	Typical KS facies, pulmonary hypertension	Unknown	5	Symptomatic	Death due to RF	[27]
Membranoproliferative GN	Typical KS facies, CVID, recurrent AOM *	Unknown	11	GCS, ARB	Improvement in both microscopic and urinary analyses	[198]
Renal agenesis	Typical KS facies, recurrent AOM, recurrent UTI	Unknown	7	Symptomatic, rehabilitation	Positive effect	[199]

Immune abnormalities associated with KS may play a role in membranoproliferative glomerulonephritis development. Urinalysis should be regularly performed in KS patients with hypogammaglobulinemia or recurrent infections [198].

## References

[1] Li Y., Bogerhausen N., Alanay Y., Kiper P.O., Plume N., Keupp K., Pohl E., Pawlik B., Rachwalski M., Milz E. (2011). A mutation screen in patients with Kabuki syndrome. Hum. Genet..

[2] Cheon C.K., Ko J.M. (2015). Kabuki syndrome: Clinical and molecular characteristics. Korean J. Pediatr..

[3] Ng S.B., Bigham A.W., Buckingham K.J., Hannibal M.C., Mcmillin M.J., Gildersleeve H.I., Beck A.E., Tabor H.K., Cooper G.M., Mefford H.C. (2011). Exome sequencing identifies MLL2 mutations as a cause of Kabuki syndrome. Nat. Genet..

[4] Bögershausen N., Wollnik B. (2013). Unmasking Kabuki syndrome. Clin. Genet..

[5] Adam M.P., Banka S., Bjornsson H.T., Bodamer O., Chudley A.E., Harris J., Kawame H., Lanpher B.C., Lindsley A.W., Merla G. (2019). Kabuki syndrome: International consensus diagnostic criteria. J. Med. Genet..

[6] Lee J.E., Wang C., Xu S., Cho Y.W., Wang L., Feng X., Baldridge A., Sartorelli V., Zhuang L., Peng W. (2013). H3K4 mono- and di-methyltransferase MLL4 is required for enhancer activation during cell differentiation. eLife.

[7] Greenfield A., Carrel L., Pennisi D., Philippe C., Quaderi N., Siggers P., Steiner K., Tam P.P.L., Willard H.F., Koopman P. (1998). The UTX gene escapes X inactivation in mice and humans. Hum. Mol. Genet..

[8] Miyake N., Mizuno S., Okamoto N., Ohashi H., Shiina M., Ogata K., Tsurusaki Y., Nakashima M., Saitsu H., Niikawa N. (2012). KDM6A Point Mutations Cause Kabuki Syndrome. Hum. Mut..

[9] Gažová I., Lengeling A., Summers K.M. (2019). Lysine demethylases KDM6A and UTY: The X and Y of histone demethylation. Mol. Genet. Metab..

[10] Bjornsson H.T., Benjamin J.S., Zhang L., Weissman J., Gerber E.E., Chen Y., Vaurio R.G., Potter M.C., Kasper D., Dietz H.C. (2014). Histone deacetylase inhibition rescues structural and functional brain deficits in a mouse model of Kabuki syndrome. Sci. Transl. Med..

[11] Dhar S.S., Lee S.H., Kan P.Y., Voigt P., Ma L., Shi X., Reinberg D., Lee M.G. (2012). Trans-tail regulation of MLL4-catalyzed H3K4 methylation by H4R3 symmetric dimethylation is mediated by a tandem PHD of MLL4. Genes Dev..

[12] Froimchuk E., Jang Y., Ge K. (2017). Histone H3 lysine 4 methyltransferase KMT2D. Gene.

[13] Schwenty-Lara J., Nehl D., Borchers A. (2020). The histone methyltransferase KMT2D, mutated in Kabuki syndrome patients, is required for neural crest cell formation and migration. Hum. Mol. Genet..

[14] Carosso G.A., Boukas L., Augustin J.J., Nguyen H.N., Winer B.L., Cannon G.H., Robertson J.D., Zhang L., Hansen K.D., Goff L.A. (2019). Precocious neuronal differentiation and disrupted oxygen responses in Kabuki syndrome. JCI Insight.

[15] Van Laarhoven P.M., Neitzel L.R., Quintana A.M., Geiger E.A., Zackai E.H., Clouthier D.E., Artinger K.B., Ming J.E., Shaikh T.H. (2015). Kabuki syndrome genes KMT2D and KDM6A: Functional analyses demonstrate critical roles in craniofacial, heart and brain development. Hum. Mol. Genet..

[16] Yamamoto P.K., de Souza T.A., Antiorio A.T.F.B., Zanatto D.A., Garcia-Gomes M.d.S.A., Alexandre-Ribeiro S.R., Oliveira N.d.S., Menck C.F.M., Bernardi M.M., Massironi S.M.G. (2019). Genetic and behavioral characterization of a Kmt2d mouse mutant, a new model for Kabuki Syndrome. Genes Brain Behav..

[17] Munehira Y., Yang Z., Gozani O. (2017). Systematic analysis of known and candidate lysine demethylases in the regulation of myoblast differentiation. J. Mol. Biol..

[18] Cocciadiferro D., Augello B., De Nittis P., Zhang J., Mandriani B., Malerba N., Squeo G.M., Romano A., Piccinni B., Verri T. (2018). Dissecting KMT2D missense mutations in Kabuki syndrome patients. Hum. Mol. Genet..

[19] Cuvertino S., Hartill V., Colyer A., Garner T., Nair N., Al-Gazali L., Canham N., Faundes V., Flinter F., Hertecant J. (2020). A restricted spectrum of missense KMT2D variants cause a multiple malformations disorder distinct from Kabuki syndrome. Genet. Med..

[20] Niikawa N., Kuroki Y., Kajii T., Matsuura N., Ishikiriyama S., Tonoki H., Ishikawa N., Yamada Y., Fujita M., Umemoto H. (1988). Kabuki make-up (Niikawa-Kuroki) syndrome: A study of 62 patients. Am. J. Med. Genet..

[21] Schrander-Stumpel C., Meinecke P., Wilson G., Gilleseen-Kaesbach G., Tinschert S., König R., Philip N., Rizzo R., Schrander J., Pfeiffer L. (1994). The Kabuki (Niikawa-Kuroki) syndrome: Further delineation of the phenotype in 29 non-Japanese patients. Eur. J. Pediatr..

[22] Wessels M.W., Brooks A.S., Hoogeboom J., Niermeijer M.F., Willems P.J. (2002). Kabuki syndrome: A review study of three hundred patients. Clin. Dysmorphol..

[23] White S.M., Thompson E.M., Kidd A., Savarirayan R., Turner A., Amor D., Delatycki M.B., Fahey M., Baxendale A., White S. (2004). Growth, behavior, and clinical findings in 27 patients with Kabuki (Niikawa-Kuroki) syndrome. Am. J. Med. Genet. Part A.

[24] Richmond E., Rogol A., Hindmarsh P.C. (2010). Current indications for growth hormone therapy. Current Indications for Growth Hormone Therapy.

[25] Schott D.A. (2018). Growth Hormone Therapy in Kabuki Syndrome: Prospective Study on the Metabolic and Longitudinal Growth Effects of rhGH Treatment in Children with Kabuki Syndrome.

[26] Ruault V., Corsini C., Duflos C., Akouete S., Georgescu V., Abaji M., Alembick Y., Alix E., Amiel J., Amouroux C. (2020). Growth charts in Kabuki syndrome 1. Am. J. Med. Genet. Part A.

[27] Armstrong L., El Moneim A.A., Aleck K., Aughton D.J., Baumann C., Braddock S.R., Gillessen-Kaesbach G., Graham J.M., Grebe T.A., Gripp K.W. (2005). Further delineation of Kabuki syndrome in 48 well-defined new individuals. Am. J. Med. Genet. Part A.

[28] Nassa G., Giurato G., Salvati A., Gigantino V., Pecoraro G., Lamberti J., Rizzo F., Nyman T.A., Tarallo R., Weisz A. (2019). The RNA-mediated estrogen receptor α interactome of hormone-dependent human breast cancer cell nuclei. Sci. Data.

[29] Schott D.A., Blok M.J., Gerver W.J.M., Devriendt K., Zimmermann L.J.I., Stumpel C.T.R.M. (2016). Growth pattern in Kabuki syndrome with a KMT2D mutation. Am. J. Med. Genet. Part A.

[30] Fahrner J.A., Lin W.Y., Riddle R.C., Boukas L., DeLeon V.B., Chopra S., Lad S.E., Luperchio T.R., Hansen K.D., Bjornsson H.T. (2019). Precocious chondrocyte differentiation disrupts skeletal growth in Kabuki syndrome mice. JCI Insight.

[31] Penders B., Schott N., Gerver W.J.M., Stumpel C.T.R.M. (2016). Body proportions in children with Kabuki syndrome. Am. J. Med. Genet. Part A.

[32] Yap K.L., Johnson A.E.K., Fischer D., Kandikatla P., Deml J., Nelakuditi V., Halbach S., Jeha G.S., Burrage L.C., Bodamer O. (2019). Congenital hyperinsulinism as the presenting feature of Kabuki syndrome: Clinical and molecular characterization of 10 affected individuals. Genet. Med..

[33] Gole H., Chuk R., Coman D. (2016). Persistent hyperinsulinism in Kabuki syndrome 2: Case report and literature review. Clin. Pract..

[34] Agger K., Cloos P.A.C., Christensen J., Pasini D., Rose S., Rappsilber J., Issaeva I., Canaani E., Salcini A.E., Helin K. (2007). UTX and JMJD3 are histone H3K27 demethylases involved in HOX gene regulation and development. Nature.

[35] Takagi M., Ishii T., Torii C., Kosaki K., Hasegawa T. (2014). A novel mutation in SOX3 polyalanine tract: A case of kabuki syndrome with combined pituitary hormone deficiency harboring double mutations in MLL2 and SOX3. Pituitary.

[36] Van Lierde K.M., Van Borsel J., Van Cauwenberge P. (2000). Speech patterns in Kabuki make-up syndrome: A case report. J. Commun. Disord..

[37] Schrander-Stumpel C.T.R.M., Spruyt L., Curfs L.M.G., Defloor T., Schrander J.J.P. (2005). Kabuki syndrome: Clinical data in 20 patients, literature review, and further guidelines for preventive management. Am. J. Med. Genet. Part A.

[38] Kondratenko I.V., Suspitsin E.N., Vakhlyarskaya S.S., Bologov A.A., Imyanitov E.N. (2017). Kabuki syndrome. Pediatr. Hematol. Immunopathol..

[39] Upton S., Stadter C.S., Landis P., Wulfsberg E.A. (2003). Speech characteristics in the Kabuki syndrome. Am. J. Med. Genet. Part A.

[40] Schott D.A., Stumpel C.T.R.M., Klaassens M. (2019). Hypermobility in individuals with Kabuki syndrome: The effect of growth hormone treatment. Am. J. Med. Genet. Part A.

[41] Vaux K.K., Jones K.L., Jones M.C., Schelley S., Hudgins L. (2005). Developmental outcome in Kabuki syndrome. Am. J. Med. Genet. Part A.

[42] van Dongen L.C.M., Wingbermühle P.A.M., van der Veld W.M., Stumpel C., Kleefstra T., Egger J.I.M. (2019). Exploring the cognitive phenotype of Kabuki (Niikawa–Kuroki) syndrome. J. Intellect. Disabil. Res..

[43] Lepri F.R., Cocciadiferro D., Augello B., Alfieri P., Pes V., Vancini A., Caciolo C., Squeo G.M., Malerba N., Adipietro I. (2018). Clinical and neurobehavioral features of three novel kabuki syndrome patients with mosaic KMT2D mutations and a review of literature. Int. J. Mol. Sci..

[44] Lehman N., Mazery A., Visier A., Baumann C., Lachesnais D., Capri Y., Toutain A., Odent S., Goizet C. (2017). Patients with Kabuki Syndrome and KMT2D Mutations. Clin. Genet..

[45] Caciolo C., Alfieri P., Piccini G., Digilio M.C., Lepri F.R., Tartaglia M., Menghini D., Vicari S. (2018). Neurobehavioral features in individuals with Kabuki syndrome. Mol. Genet. Genom. Med..

[46] Parisi L., Di Filippo T., Roccella M. (2015). Autism spectrum disorder in Kabuki syndrome: Clinical, diagnostic and rehabilitative aspects assessed through the presentation of three cases. Minerva Pediatr..

[47] Rangasamy S., D’Mello S.R., Narayanan V. (2013). Epigenetics, Autism Spectrum, and Neurodevelopmental Disorders. Neurotherapeutics.

[48] Benjamin J.S., Pilarowski G.O., Carosso G.A., Zhang L., Huso D.L., Goff L.A., Vernon H.J., Hansen K.D., Bjornsson H.T. (2017). A ketogenic diet rescues hippocampal memory defects in a mouse model of Kabuki syndrome. Proc. Natl. Acad. Sci. USA.

[49] Hannibal M.C. Hypoglycemia—How It Relates to Kabuki Syndrome. https://kabukisyndrome.com/content/hypoglycemia-how-it-relates-kabuki-syndrome#:~:text=Glucoseistheprimaryfuel,hypoglycemiathroughoutinfancyandchildhood.

[50] Verrotti A., Agostinelli S., Cirillo C., D’Egidio C., Mohn A., Boncimino A., Coppola G., Spalice A., Nicita F., Pavone P. (2011). Long-term outcome of epilepsy in Kabuki syndrome. Seizure.

[51] Kurahashi N., Miyake N., Mizuno S., Koshimizu E., Kurahashi H., Yamada K., Natsume J., Aoki Y., Nakamura M., Taniai H. (2017). Characteristics of epilepsy in patients with Kabuki syndrome with KMT2D mutations. Brain Dev..

[52] Ogawa A., Yasumoto S., Tomoda Y., Ohfu M., Mitsudome A., Kuroki Y. (2003). Favorable seizure outcome in Kabuki make-up syndrome associated with epilepsy. J. Child Neurol..

[53] Di Gennaro G., Condoluci C., Casali C., Ciccarelli O., Albertini G. (1999). Epilepsy and polymicrogyria in Kabuki make-up (Niikawa-Kuroki) syndrome. Pediatr. Neurol..

[54] Mihçi E., Taçoy S., Haspolat S., Karaali K. (2002). Central nervous system abnormalities in Kabuki (Niikawa-Kuroki) syndrome [4]. Am. J. Med. Genet..

[55] Powell H.W.R., Hart P.E., Sisodiya S.M. (2003). Epilepsy and perisylvian polymicrogyria in a patient with Kabuki syndrome. Dev. Med. Child Neurol..

[56] Topcu Y., Bayram E., Karaoglu P., Yis U., Kabuki S.H.K. (2013). syndrome and perisylvian cortical dysplasia in a Turkish girl. J. Pediatr. Neurosci..

[57] Lodi M., Chifari R., Parazzini C., Viri M., Beccaria F., Lorenzetti M.E., Meloni M., Capovilla G., Romeo A. (2010). Seizures and EEG pattern in Kabuki syndrome. Brain Dev..

[58] Bekircan-Kurt C.E., Şimşek-Kiper P.Ö., Boduroğlu K., Dericioğlu N. (2016). A novel de novo mutation involving the MLL2 gene in a Kabuki syndrome patient presenting with seizures. Turk. J. Pediatr..

[59] Ciprero K.L., Clayton-Smith J., Donnai D., Zimmerman R.A., Zackai E.H., Ming J.E. (2005). Symptomatic Chiari I malformation in Kabuki syndrome. Am. J. Med. Genet. Part A.

[60] Greenlee J.D.W., Donovan K.A., Hasan D.M., Menezes A.H. (2002). Chiari I malformation in the very young child: The spectrum of presentations and experience in 31 children under age 6 years. Pediatrics.

[61] Topa A., Samuelsson L., Lovmar L., Stenman G., Kölby L. (2017). On the significance of craniosynostosis in a case of Kabuki syndrome with a concomitant KMT2D mutation and 3.2 Mbp de novo 10q22.3q23.1 deletion. Am. J. Med. Genet. Part A.

[62] Zollino M., Lattante S., Orteschi D., Frangella S., Doronzio P.N., Contaldo I., Mercuri E., Marangi G. (2017). Syndromic craniosynostosis can define new candidate genes for suture development or result from the non-specifc effects of pleiotropic genes: Rasopathies and chromatinopathies as examples. Front. Neurosci..

[63] Marzollo A., Colavito D., Sartori S., Fanelli G.N., Putti M.C. (2018). Cerebral Lymphoproliferation in a Patient with Kabuki Syndrome. J. Clin. Immunol..

[64] Gidwani P., Segal E., Shanske A., Driscoll C. (2007). Chorea Associated with Antiphospholipid Antibodies in a Patient with Kabuki Syndrome. Am. J. Med. Genet..

[65] Boisgontier J., Tacchella J.M., Lemaître H., Lehman N., Saitovitch A., Gatinois V., Boursier G., Sanchez E., Rechtman E., Fillon L. (2019). Anatomical and functional abnormalities on MRI in kabuki syndrome. NeuroImage Clin..

[66] Grunseich C., Fishbein T.M., Berkowitz F., Shamim E.A. (2010). Tremor and Deep Brain Nuclei Hyperintensities in Kabuki Syndrome. Pediatr. Neurol..

[67] Kasdon B.D., Fox J.E. (2012). Kabuki syndrome: Diagnostic and treatment considerations. Ment. Health Fam. Med..

[68] Digilio M.C., Marino B., Toscano A., Giannotti A., Dallapiccola B. (2001). Congenital heart defects in Kabuki syndrome. Am. J. Med. Genet..

[69] Mcmahon C.J., Reardon W. (2006). The spectrum of congenital cardiac malformations encountered in six children with Kabuki syndrome. Cardiol. Young.

[70] Digilio M.C., Gnazzo M., Lepri F., Dentici M.L., Pisaneschi E., Baban A., Passarelli C., Capolino R., Angioni A., Novelli A. (2017). Congenital heart defects in molecularly proven Kabuki syndrome patients. Am. J. Med. Genet. Part A.

[71] Hughes H.E. (1994). Coarctation of the aorta in Kabuki syndrome. Arch. Dis. Child..

[72] Bhat A.H., Davenport J., Cocalis M. (2012). Partial Anomalous Left Pulmonary Artery along with Aortic Coarctation in an Infant with Kabuki Syndrome. Echocardiography.

[73] Ko H., Kim G., Lee H.D., Kim H., Sung S.C. (2019). Coarctation of the aorta and left ventricular diverticulum in Kabuki syndrome. Pediatr. Int..

[74] Ang S., Uebersohn A., Spencer C.I., Huang Y., Lee J., Ge K. (2016). KMT2D regulates specific programs in heart development via histone H3 lysine 4 di-methylation. Development.

[75] Stangler Herodez S., Marcun Varda N., Kokalj Vokac N., Krgovic D. (2020). De Novo KMT2D heterozygous frameshift deletion in a newborn with a congenitcal heart anomaly. BJMG.

[76] Schwenty-Lara J., Nurnberger A., Borchers A. (2019). Loss of function of Kmt2d, a gene mutated in Kabuki syndrome, affects heart development in Xenopus laevis. Dev. Dyn..

[77] Serrano M.A., Demarest B.L., Tone-Pah-Hote T., Tristani-Firouzi M., Yost H.J. (2019). Inhibition of notch signaling rescues cardiovascular development in Kabuki syndrome. PLoS Biol..

[78] Shahdadpuri R., Ann S., Mcmahon H., McMahon C.J. (2008). A Novel Constellation of Cardiac Findings for Kabuki Syndrome: Hypoplastic Left Heart Syndrome and Partial Anomalous Pulmonary Venous Drainage. Pediatric Cardiol..

[79] Onan B., Kahraman Z. (2017). Robotic surgery for atrial septal defect closure in a case of Kabuki syndrome. Arch. Turk. Soc. Cardiol..

[80] Rao A.S., Makaroun M.S., Marone L.K., Cho J.S., Rhee R., Chaer R.A. (2011). Long-term outcomes of internal carotid artery dissection. J. Vasc. Surg..

[81] Dyamenahalli U., Abraham B., Fontenot E., Prasad V., Imamura M. (2007). Pathologic Aneurysmal Dilation of the Ascending Aorta and Dilation of the Main Pulmonary Artery in Patients with Kabuki. Congenit. Heart Dis..

[82] Moral S., Zuccarino F., Loma-Osorio P. (2009). Double aortic arch: An unreported anomaly with Kabuki syndrome. Pediatr. Cardiol..

[83] Blum K.A., Yaghi S. (2016). 2016 ICSWP Guidelines. Arch. Neurosci..

[84] Yoon J.K., Ahn K.J., Kwon B.S., Kim G.B., Bae E.J., Noh C.I., Ko J.M. (2015). The strong association of left-side heart anomalies with Kabuki syndrome. Korean J. Pediatrics.

[85] Kung G.C., Chang P.M., Sklansky M.S., Randolph L.M. (2010). Hypoplastic Left Heart Syndrome in Patients with Kabuki Syndrome. Pediatric Cardiol..

[86] Shah M., Bogucki B., Mavers M., Daphne E., Knutsen A. (2005). BMC Medical Genetics Cardiac conduction abnormalities and congenital immunodeficiency in a child with Kabuki syndrome: Case report. BMC Med. Genet..

[87] Kawame H., Hannibal M.C., Hudgins L., Pagon R.A. (1999). Phenotypic spectrum and management issues in Kabuki syndrome. J. Pediatr..

[88] Dentici M.L., Di Pede A., Lepri F.R., Gnazzo M., Lombardi M.H., Auriti C., Petrocchi S., Pisaneschi E., Bellacchio E., Capolino R. (2015). Kabuki syndrome: Clinical and molecular diagnosis in the first year of life. Arch. Dis. Child..

[89] Siminas S., Baillie C., Turnock R. (2015). Kabuki Syndrome and Anorectal Malformations: Implications for Diagnosis and Treatment. Eur. J. Pediatr. Surg. Reports.

[90] Bakhit M., Kowalski T. (2020). Interconnecting pancreatic ducts: Unique ductogenesis in a patient with Kabuki syndrome and cytochrome C deficiency. Gastrointest. Endosc..

[91] Diaconescu S., Miron I., Gimiga N., Olaru C., Ioniuc I., Ciongradi I., Sarbu I., Stefanescu G. (2016). Unusual endoscopic findings in children: Esophageal and gastric polyps: Three cases report. Medicine.

[92] Suskind D.L., Finn L., Wahbeh G., Christie D., Horslen S. (2006). A child with kabuki syndrome and primary sclerosing cholangitis successfully treated with ursodiol and cholestryamine. J. Pediatr. Gastroenterol. Nutr..

[93] Timothy L.D., Lehrke H.D., Chandan V.S., Kolbe A.B., Furuya K.N. (2019). Diffuse Adenomatosis and Hepatocellular Carcinoma Treated with Liver Transplantation in an Adolescent Female with Kabuki Syndrome with a Novel KMT2D Gene Mutation. Case Rep. Pediatr..

[94] Ming J.E., Russell K.L., McDonald-McGinn D.M., Zackai E.H. (2005). Autoimmune disorders in Kabuki syndrome. Am. J. Med. Genet. Part A.

[95] Ho J., Fox D., Innes A.M., McLeod R., Butzner D., Johnson N., Trevenen C., Kendrick V., Cole D.E.C. (2010). Kabuki syndrome and Crohn disease in a child with familial hypocalciuric hypercalcemia. J. Pediatr. Endocrinol. Metab..

[96] Barozzi S., Di Berardino F., Atzeri F., Filipponi E., Cerutti M., Selicorni A., Cesarani A. (2009). Audiological and vestibular findings in the Kabuki syndrome. Am. J. Med. Genet. Part A.

[97] Fong C.T., Wang M., Young E.C., Hogan C.A., Tallents R.H., Kyrkanides S., Liptak G.S., Sanger J.A., Frisina R.D. (2001). Microtia associated with the Kabuki (Niikawa-Kuroki) syndrome. Otolaryngol.—Head Neck Surg..

[98] Igawa H.H., Nishizawa N., Sugihara T., Inuyama Y. (2000). Inner Ear Abnormalities in Kabuki Make-Up Syndrome: Report of Three Cases. Am. J. Med. Genet..

[99] David-Paloyo F.P., Yang X., Lin J.L., Wong F.H., Wu-Chou Y.H., Lo L.J. (2014). Lower Lip Pits: Van der Woude or Kabuki Syndrome?. Cleft Palate-Craniofacial J..

[100] Peterson-Falzone S.J., Golabi M., Lalwani A.K. (1997). Otolaryngologic manifestations of Kabuki syndrome. Int. J. Pediatr. Otorhinolaryngol..

[101] Defloor T., Van Borsel J., Schrander-Stumpel C.T.R.M., Curfs L.M.G. (2005). Expressive Language in Children with Kabuki Syndrome Mean Length of Utterance. Am. J. Med. Genet. Part A.

[102] Tekin M., Fitoz S., Arici S., Cetinkaya E. (2006). Niikawa—Kuroki (Kabuki) syndrome with congenital sensorineural deafness: Evidence for a wide spectrum of inner ear abnormalities. Int. J. Pediatr. Otorhinolaryngol..

[103] Toutain A., Plee Y., Ployet M., S B., Perrot A., Sembely C., Barthez M., Morraine C. (1997). Deafness and Mondini dysplasia in Kabuki make-up syndrome: Report of a case and review of the literature. Genet. Couns..

[104] Lin J., Lee W., Huang J., Chen P.K., Chan K. (2015). Immunologic Assessment and KMT2D mutation detection in Kabuki Syndrome. Clin. Genet..

[105] Pepe G., Negri M., Falcioni M., Di Lella F., Vincenti V. (2020). Bonebridge implantation for mixed hearing loss in a patient with Kabuki syndrome. Acta Bio Med. Atenei Parm..

[106] Lu Y., Cao K. (2014). ScienceDirect A case report: Hearing disorder in Kabuki make-up ( Niikawa-Kuroki ) syndrome in China. J. Otol..

[107] Petzold D., Kratzsch E., Opitz C., Tinschert S. (2003). The Kabuki syndrome: Four patients with oral abnormalities. Eur. J. Orthod..

[108] Shpargel K.B., Starmer J., Wang C., Ge K., Magnuson T. (2017). UTX-guided neural crest function underlies craniofacial features of Kabuki syndrome. Proc. Natl. Acad. Sci. USA.

[109] Shpargel K.B., Mangini C.L., Xie G., Ge K., Magnuson T. (2020). The KMT2D Kabuki syndrome histone methylase controls neural crest cell differentiation and facial morphology. Development.

[110] do Prado Sobral S., Leite A.F., Figueiredo P.T.S., Ferrari I., Safatle H.P.N., Cordoba M.S., Versiani B.R., Acevedo A.C., Mestrinho H.D. (2013). Craniofacial and Dental Features in Kabuki Syndrome Patients. Cleft Palate-Craniofacial J..

[111] Paik J.M., Lim S.Y. (2016). Kabuki Syndrome with Cleft Palate. Arch. Plast. Surg..

[112] Iida T., Park S., Kato K., Kitano I. (2006). Cleft Palate in Kabuki Syndrome: A Report of Six Cases. Cleft Palate-Craniofacial J..

[113] Porntaveetus T., Abid M.F., Theerapanon T., Srichomthong C. (2018). Expanding the Oro—Dental and Mutational Spectra of Kabuki Syndrome and Expression of KMT2D and KDM6A in Human Tooth Germs. Int. J. Biol. Sci..

[114] Tuna E., Marsan G., Gencay K., Seymen F. (2012). Craniofacial and Dental Characteristics of Kabuki Syndrome: Nine Years Cephalometric Follow-Up. J. Clin. Pediatr. Dent..

[115] Matsune K., Shimizu T., Tohma T., Asada Y., Ohashi H., Maeda T. (2001). Craniofacial and Dental Characteristics of Kabuki Syndrome. Am. J. Med. Genet..

[116] Silva-andrade N., López-ortega K., Gallottini M. (2019). Orofacial features and medical profile of eight individuals with Kabuki syndrome. Med. Oral Patol. Oral Cir. Bucal.

[117] Cogulu D., Celen E., Oncag O., Ozkinay F. (2008). Kabuki Syndrome with Additional Dental Findings: A Case Report. J. Dent. Child..

[118] Atar M., Lee W. (2006). Kabuki syndrome: Oral and general features seen in a 2-year-old Chinese boy. Int. J. Paediatr. Dent..

[119] Teixeira C., Silva C., Honjo R., Bertola D., Albano L., Kim C. (2009). Dental Evaluation of Kabuki Syndrome Patients. Cleft Palate-Craniofacial J..

[120] Cudzilo D., Czochrowska E. (2018). Orthodontic Treatment of a Kabuki Syndrome Patient. Cleft Palate-Craniofacial J..

[121] Burke L.W., Jones M.C. (1995). Kabuki syndrome: Underdiagnosed recognizable pattern in cleft palate patients. Cleft Palate-Craniofacial J..

[122] Abdel-Salam G., Afifi H., Eid M., El-Badry T., Kholoussi N. (2008). Anorectal anomalies, diaphragmatic defect, cleft palate, lower lip pits, hypopigmentation and hypogammaglobulinemia A in Kabuki syndrome: A rare combination. Genet. Couns..

[123] Cheon C.K., Choi H.Y., Park S.H., Jung J.H., Kim S.J. (2020). Ocular manifestations in kabuki syndrome: A report of 10 cases and literature review. Ophthalmic Genet..

[124] Evans S.L., Kumar N., Rashid M.H., Hughes D.S. (2004). New ocular findings in a case of Kabuki syndrome. Eye.

[125] Turner C.L.S., Lachlan K., Amerasinghe N., Hodgkins P., Maloney V., Barber J., Temple I.K. (2005). Kabuki syndrome: New ocular findings but no evidence of 8p22-p23.1 duplications in a clinically defined cohort. Eur. J. Hum. Genet..

[126] Sharma P., Dave V. (2010). Esotropia in Kabuki syndrome. J. Pediatr. Ophthalmol. Strabismus.

[127] Hamdi T., Basheikh A. (2020). Large Angle Congenital Esotropia in a Child With Kabuki Syndrome: A Case Report. Cureus.

[128] Onwochei B.C., Simon J.W., Bateman J.B., Couture K.C., Mir E. (2000). Ocular Colobomata. Surv. Ophthalmol..

[129] Ming J.E., Russell K.L., Bason L., McDonald-McGinn D.M., Zackai E.H. (2003). Coloboma and Other Ophthalmologic Anomalies in Kabuki Syndrome: Distinction from Charge Association. Am. J. Med. Genet. Part A.

[130] Verhagen J.M.A., Oostdijk W., Terwisscha van Scheltinga C.E.J., Schalij-Delfos N.E., van Bever Y. (2014). An unusual presentation of Kabuki syndrome: Clinical overlap with CHARGE syndrome. Eur. J. Med. Genet..

[131] Tanaka R., Takenouchi T., Uchida K., Sato T., Fukushima H., Yoshihashi H., Takahashi T., Tsubota K., Kosaki K. (2012). Congenital corneal staphyloma as a complication of Kabuki syndrome. Am. J. Med. Genet. Part A.

[132] Lin P.A., Tseng S.H., Lai I.W., Huang Y.H. (2019). Bilateral Congenital Corneal Opacities as an Early-Onset Ocular Feature of Kabuki Syndrome. Cornea.

[133] Nischal K.K., Naor J., Jay V., MacKeen L.D., Rootman D.S. (2002). Clinicopathological correlation of congenital corneal opacification using ultrasound biomicroscopy. Br. J. Ophthalmol..

[134] Diez M.T.S., Lemaitre S., Gonzalez-Valdivia H., Gonzalez-Candial M.G. (2020). Lacrimal puncta agenesis in kabuki syndrome. Ophthal. Plast. Reconstr. Surg..

[135] Sakurai H., Nozaki M., Takcuchi M., Socjima K., Kajimoto M., Hori S. (2003). Periorbital corection in Kabuki Syndrome. Plast. Reconstr. Surg..

[136] McVeigh T.P., Banka S., Reardon W. (2015). Kabuki syndrome: Expanding the phenotype to include microphthalmia and anophthalmia. Clin. Dysmorphol..

[137] Emmert-buck L.T., Preslan M.W., Kathuria S.S. (2004). Jaw-Winking Ptosis in a Patient with Kabuki Syndrome. J. Pediatr. Ophthalmol. Strabismus.

[138] Martins A., Oliveira M.A., Rosa A., Murta J. (2019). Salzmann nodular degeneration features in a case of Kabuki make-up syndrome. BMJ Case Rep..

[139] Chen Y.H., Sun M.H., Hsia S.H., Lai C.C., Wu W.C. (2014). Rare ocular features in a case of Kabuki syndrome (Niikawa-Kuroki syndrome). BMC Ophthalmol..

[140] Bravetti G.E., Gillmann K., Mansouri K., Mermoud A. (2019). Congenital Refractory Glaucoma: A New Ophthalmic Association of Kabuki Syndrome and its Management with Glaucoma Drainage Devices. J. Glaucoma.

[141] Chuah J.L., Chuah J.K., Brown R. (2009). New fundus findings in a case of Kabuki syndrome. Eye.

[142] Lindfield D., Griffiths M.F.P., Thompson D.A., Moore A.T. (2011). Macular dystrophy in Kabuki syndrome: A new clinical feature?. J. Pediatr. Ophthalmol. Strabismus.

[143] Elsherbiny S.M. (2002). Macular deposits: A new feature of Kabuki syndrome?. J. Pediatr. Ophthalmol. Strabismus.

[144] del Cerro I., Merino P., Gómez de Liaño P., Alan G. (2020). Changes in ocular motility in Kabuki syndrome. Arch. Soc. Esp. Oftalmol..

[145] Wang Y.R., Xu N.X., Wang J., Wang X.M. (2019). Kabuki syndrome: Review of the clinical features, diagnosis and epigenetic mechanisms. World J. Pediatr..

[146] Alam H., Tang M., Maitituoheti M., Dhar S.S., Kumar M., Han C.Y., Ambati C.R., Amin S.B., Gu B., Chen T.Y. (2020). KMT2D Deficiency Impairs Super-Enhancers to Confer a Glycolytic Vulnerability in Lung Cancer. Cancer Cell.

[147] Faundes V., Malone G., Newman W.G., Banka S. (2019). A comparative analysis of KMT2D missense variants in Kabuki syndrome, cancers and the general population. J. Hum. Genet..

[148] Guo C., Chen L.H., Huang Y., Chang C.C., Wang P., Pirozzi C.J., Qin X., Bao X., Greer P.K., McLendon R.E. (2013). KMT2D maintains neoplastic cell proliferation and global histone H3 lysine 4 monomethylation. Oncotarget.

[149] Parsons D.W., Li M., Zhang X., Jones S., Leary R.J., Lin J.C., Boca S.M., Carter H., Samayoa J., Gallia G.L. (2011). The genetic landscape of medulloblastoma. Science.

[150] Smith E., Lin C., Shilatifard A. (2011). The super elongation complex (SEC) and MLL in development and disease. Genes Dev..

[151] Tran N., Broun A., Ge K. (2020). Lysine Demethylase KDM6A in Differentiation, Development, and Cancer. Mol. Cell. Biol..

[152] Van Haaften G., Dalgliesh G.L., Davies H., Chen L., Bignell G., Greenman C., Edkins S., Hardy C., O’Meara S., Teague J. (2009). Somatic mutations of the histone H3K27 demethylase gene UTX in human cancer. Nat. Genet..

[153] Van Der Meulen J., Speleman F., Van Vlierberghe P. (2014). The H3K27me3 demethylase UTX in normal development and disease. Epigenetics.

[154] Teranishi H., Koga Y., Nakashima K., Morihana E., Ishii K., Sakai Y., Taguchi T., Oda Y., Miyake N., Matsumoto N. (2018). Cancer management in kabuki syndrome: The first case of wilms tumor and a literature review. J. Pediatr. Hematol. Oncol..

[155] Tumino M., Licciardello M., Sorge G., Cutrupi M.C., Di Benedetto F., Amoroso L., Catania R., Pennisi M., D’Amico S., Di Cataldo A. (2010). Kabuki syndrome and cancer in two patients. Am. J. Med. Genet. Part A.

[156] Merks J.H.M., Caron H.N., Hennekam R.C.M. (2005). High incidence of malformation syndromes in a series of 1,073 children with cancer. Am. J. Med. Genet. Part A.

[157] Shahdadpuri R., O’Meara A., O’Sullivan M., Reardon W. (2008). Low-grade fibromyxoid sarcoma: Yet another malignancy associated with Kabuki syndrome. Clin. Dysmorphol..

[158] Scala M., Morana G., Sementa A.R., Merla G., Piatelli G., Capra V., Pavanello M. (2019). Aggressive desmoid fibromatosis in Kabuki syndrome: Expanding the tumor spectrum. Pediatr. Blood Cancer.

[159] Darbari A., Sabin K.M., Shapiro C.N., Schwarz K.B. (2003). Epidemiology of primary hepatic malignancies in U.S. children. Hepatology.

[160] Cleary S.P., Jeck W.R., Zhao X., Chen K., Selitsky S.R., Gleb L., Li J., Powers S., Kim H., Fischer S. (2013). Identification of Driver Genes in Hepatocellular Carcinoma by Exome Sequencing. Hepatology.

[161] Kim D.H., Rhee J.C., Yeo S., Shen R., Lee S.K., Lee J.W., Lee S. (2015). Crucial roles of mixed-lineage leukemia 3 and 4 as epigenetic switches of the hepatic circadian clock controlling bile acid homeostasis in mice. Hepatology.

[162] Richet C., Maza A., Dreyfus I., Bourrat E., Mazereeuw-Hautier J. (2018). Childhood pilomatricomas: Associated anomalies. Pediatr. Dermatol..

[163] Bernier F.E., Schreiber A., Coulombe J., Hatami A., Marcoux D. (2017). Pilomatricoma Associated with Kabuki Syndrome. Pediatr. Dermatol..

[164] Hamahata A., Kamei W., Ishikawa M., Konoeda H., Yamaki T., Sakurai H. (2013). Multiple pilomatricomas in Kabuki syndrome. Pediatr. Dermatol..

[165] Cuesta L., Betlloch I., Toledo F., Latorre N., Monteagudo A.F. (2011). Kabuki syndrome: A new case associated with Becker nevus. Dermatol. Online J..

[166] Karagianni P., Lambropoulos V., Stergidou D., Fryssira H., Chatziioannidis I., Spyridakis I. (2016). Recurrent giant cell fibroblastoma: Malignancy predisposition in Kabuki syndrome revisited. Am. J. Med. Genet. Part A.

[167] Roma D., Palma P., Capolino R., Figà-Talamanca L., Diomedi-Camassei F., Lepri F.R., Digilio M.C., Marras C.E., Messina R., Carai A. (2015). Spinal ependymoma in a patient with Kabuki syndrome: A case report. BMC Med. Genet..

[168] Ortega-Molina A., Boss I.W., Canela A., Pan H., Jiang Y., Zhao C., Jiang M., Hu D., Agirre X., Niesvizky I. (2015). The histone lysine methyltransferase KMT2D sustains a gene expression program that represses B cell lymphoma development. Nat. Med..

[169] Kaiwar C., Kruisselbrink T.M., Kudva Y.C., Klee E.W., Pichurin P. (2019). Exome sequencing confirms diagnosis of kabuki syndrome in an-adult with hodgkin lymphoma and unusually severe multisystem phenotype. Clin. Immunol..

[170] Scherer S., Theile U., Beyer V., Ferrari R., Kreck C., Rister M. (2003). Patient with Kabuki syndrome and acute leukemia. Am. J. Med. Genet. Part A.

[171] Cheon C.K., Sohn Y.B., Ko J.M., Lee Y.J., Song J.S., Moon J.W., Yang B.K., Ha I.S., Bae E.J., Jin H.S. (2014). Identification of KMT2D and KDM6A mutations by exome sequencing in Korean patients with Kabuki syndrome. J. Hum. Genet..

[172] Ijichi O., Kawakami K., Matsuda Y., Ikarimoto N., Miyata K., Takamatsu H., Tokunaga M. (1996). A case of Kabuki make-up syndrome with EBV+ Burkitt’s lymphoma. Pediatr. Int..

[173] de Billy E., Strocchio L., Cacchione A., Agolini E., Gnazzo M., Novelli A., De Vito R., Capolino R., Digilio M.C., Caruso R. (2019). Burkitt lymphoma in a patient with Kabuki syndrome carrying a novel KMT2D mutation. Am. J. Med. Genet. Part A.

[174] Lindsley A.W., Saal H.M., Burrow T.A., Hopkin R.J., Shchelchkov O., Khandelwal P., Xie C., Bleesing J., Filipovich L., Risma K. (2016). Defects of B Cell Terminal Differentiation in Patients with Type-1 Kabuki Syndrome. J. Allergy Clin. Immunol..

[175] Pilarowski G.O., Cazares T., Zhang L., Benjamin J.S., Liu K., Jagannathan S., Mousa N., Kasten J., Barski A., Lindsley A.W. (2020). Abnormal Peyer patch development and B-cell gut homing drive IgA deficiency in Kabuki syndrome. J. Allergy Clin. Immunol..

[176] Margot H., Boursier G., Duflos C., Sanchez E., Amiel J., Andrau J.C., Arpin S., Brischoux-Boucher E., Boute O., Burglen L. (2020). Immunopathological manifestations in Kabuki syndrome: A registry study of 177 individuals. Genet. Med..

[177] Torii Y., Yagasaki H., Tanaka H., Mizuno S., Nishio N., Muramatsu H., Hama A., Takahashi Y., Kojima S. (2009). Successful treatment with rituximab of refractory idiopathic thrombocytopenic purpura in a patient with Kabuki syndrome. Int. J. Hematol..

[178] Giordano P., Lassandro G., Sangerardi M., Faienza M.F., Valente F., Martire B. (2014). Autoimmune haematological disorders in two Italian children with Kabuki syndrome. Ital. J. Pediatr..

[179] Cantoni S., Fattizzo B. (2019). Clinical course and management of adult-onset immune-mediated cytopenia associated with Kabuki syndrome. Eur. J. Intern. Med..

[180] Warnatz K., Voll R.E. (2012). Pathogenesis of autoimmunity in common variable immunodeficiency. Front. Immunol..

[181] Stagi S., Gulino A.V., Lapi E., Rigante D. (2016). Epigenetic control of the immune system: A lesson from Kabuki syndrome. Immunol. Res..

[182] Chrzanowska K., Krajewska-Walasek M., Kus J., Michalkiewicz J., Maziarka D., Wolski J., Brecevic L., Madalinski K. (1998). Kabuki (Niikawa-Kuroki) syndrome associated with immunodeficiencv. Clin. Genet..

[183] Hoffman J.D., Ciprero K.L., Sullivan K.E., Kaplan P.B., McDonald-McGinn D.M., Zackai E.H., Ming J.E. (2005). Immune abnormalities are a frequent manifestation of Kabuki syndrome. Am. J. Med. Genet. Part A.

[184] Deyà-Martínez A., Esteve-Solé A., Vélez-Tirado N., Celis V., Costa J., Cols M., Jou C., Vlagea A., Plaza-Martin A.M., Juan M. (2018). Sirolimus as an alternative treatment in patients with granulomatous-lymphocytic lung disease and humoral immunodeficiency with impaired regulatory T cells. Pediatr. Allergy Immunol..

[185] Arsov T., Sestan M., Cekada N., Frkovic M., Andrews D., He Y., Shen N., Vinuesa C.G., Jelusic M. (2019). Systemic lupus erythematosus: A new autoimmune disorder in Kabuki syndrome. Eur. J. Med. Genet..

[186] Almécija A.C., Pérez V., Baro M., Guerra-García P., Vivanco J.L. (2019). Atypical Autoimmune Hematologic Disorders in a Patient with Kabuki Syndrome. J. Pediatr. Hematol. Oncol..

[187] Zimmermann T., Brasch F., Rauch A., Stachel D., Holter W., Beck J. (2006). Lymphoid interstitial pneumonia and Kabuki- Syndrome in a young man. Paediatr. Respir. Rev..

[188] Frans G., Meyts I., Devriendt K., Liston A., Vermeulen F., Bossuyt X. (2016). Mild humoral immunodeficiency in a patient with X-linked Kabuki syndrome. Am. J. Med. Genet. Part A.

[189] Courcet J.B., Faivre L., Michot C., Burguet A., Perez-Martin S., Alix E., Amiel J., Baumann C., Cordier M.P., Cormier-Daire V. (2013). Clinical and molecular spectrum of renal malformations in kabuki syndrome. J. Pediatr..

[190] Hannibal M.C., Buckingham K.J., Ng S.B., Ming J.E., Anita E., Mcmillin M.J., Gildersleeve H.I., Bigham A.W., Tabor H.K., Mefford H.C. (2011). Spectrum of MLL2 (ALR) mutations in 110 cases of Kabuki syndrome. Am. J. Med. Genet. Part A.

[191] Ewart-Toland A., Enns G.M., Cox V.A., Chandra Mohan G., Rosenthal P., Golabi M. (1998). Severe congenital anomalies requiring transplantation in children with Kabuki syndrome. Am. J. Med. Genet..

[192] Hamdi Kamel M., Gilmartin B., Mohan P., Hickey D.P. (2006). Successful long-term outcome of kidney transplantation in a child with Kabuki syndrome. Pediatr. Transplant..

[193] Au P.Y.B., Goedhart C., Ferguson M., Breckpot J., Devriendt K., Wierenga K., Fanning E., Grange D.K., Graham G.E., Galarreta C. (2018). Phenotypic spectrum of Au–Kline syndrome: A report of six new cases and review of the literature. Eur. J. Hum. Genet..

[194] Kohei N., Takada H., Hishiki K., Nakashima Y., Yoshimura K., Nishio Y., Ito K., Matsuo K., Mori N. (2016). Spontaneous infection of an atrophic ureter with an ectopic ureteral opening after living-donor renal transplantation in a patient with kabuki syndrome. Acta Urol. Jpn..

[195] Suarez Guerrero J.L., Ordónez Suarez A.A., Contreras García G.A. (2012). Síndrome de Kabuki. An. Pediatr..

[196] Shawky R.M., Gamal R., Mostafa N. (2017). Kabuki make-up syndrome with genitourinary anomalies, ophthalmologic features and hyperpigmentation in an Egyptian child. Egypt. J. Med. Hum. Genet..

[197] Galán-Gómez E., Carbonell-Pérez J.M., Cardesa-García J.J., Val-Sánchez De León J.M., Campo-Sampedro F.M., Martínez-Frías M.L., Frías J.L. (2004). A Diagnostic Conundrum: Two Siblings with Features Overlapping the Kabuki and Malpuech Syndromes. A New MCA Syndrome?. Am. J. Med. Genet. Part A.

[198] Nishizaki N., Fujinaga S., Hirano D., Murakami H., Kamei K., Ohtomo Y., Shimizu T., Kaneko K. (2014). Membranoproliferative glomerulonephritis type 3 associated with Kabuki syndrome. Clin. Nephrol..

[199] Rosti R.Ö., Kayserili H. (2009). Kabuki make-up syndrome with unilateral renal agenesis. Turk. J. Pediatr..

